# Conversionless efficient and broadband laser light diffusers for high brightness illumination applications

**DOI:** 10.1038/s41467-020-14875-z

**Published:** 2020-03-18

**Authors:** Fabian Schütt, Maximilian Zapf, Stefano Signetti, Julian Strobel, Helge Krüger, Robert Röder, Jürgen Carstensen, Niklas Wolff, Janik Marx, Tian Carey, Marleen Schweichel, Maik-Ivo Terasa, Leonard Siebert, Hyo-Ki Hong, Sören Kaps, Bodo Fiedler, Yogendra Kumar Mishra, Zonghoon Lee, Nicola M. Pugno, Lorenz Kienle, Andrea C. Ferrari, Felice Torrisi, Carsten Ronning, Rainer Adelung

**Affiliations:** 1 Functional Nanomaterials, Institute for Materials Science, Kiel University, Kaiserstr. 2, 24143 Kiel, Germany; 2 Institute for Solid State Physics, Friedrich-Schiller-University Jena, Max-Wien-Platz 1, 07743 Jena, Germany; 3 Laboratory of Bio-inspired, Bionic, Nano, Meta Materials & Mechanics, Department of Civil, Environmental and Mechanical Engineering, University of Trento, via Mesiano 77, I-38123 Trento, Italy; 4 Synthesis and Real Structure, Institute for Materials Science, Kiel University, Kaiserstr. 2, 24143 Kiel, Germany; 5 Institute of Polymers and Composites, Hamburg University of Technology, Denickestr. 15, 21073 Hamburg, Germany; 6 Cambridge Graphene Centre, University of Cambridge, 9, JJ Thomson Avenue, Cambridge, CB3 0FA UK; 7 Department of Chemistry, Molecular Sciences Research Hub, Imperial College London, White City Campus, Wood Lane, London, W12 0BZ UK; 8 School of Materials Science and Engineering, Ulsan National Institute of Science and Technology (UNIST), Ulsan, 44919 Republic of Korea; 9 SDU NanoSYD, Mads Clausen Institute, University of Southern Denmark, Alsion 2, 6400 Sønderborg, Denmark; 10 Center for Multidimensional Carbon Materials, Institute for Basic Science (IBS), Ulsan, 44919 Republic of Korea; 11 School of Engineering and Materials Science, Queen Mary University of London, Mile End Road E1 4NS, London, UK; 12 Ket-Lab, Edoardo Amaldi Foundation, via del Politecnico snc, I-00133 Roma, Italy

**Keywords:** Lasers, LEDs and light sources, Two-dimensional materials

## Abstract

Laser diodes are efficient light sources. However, state-of-the-art laser diode-based lighting systems rely on light-converting inorganic phosphor materials, which strongly limit the efficiency and lifetime, as well as achievable light output due to energy losses, saturation, thermal degradation, and low irradiance levels. Here, we demonstrate a macroscopically expanded, three-dimensional diffuser composed of interconnected hollow hexagonal boron nitride microtubes with nanoscopic wall-thickness, acting as an artificial solid fog, capable of withstanding ~10 times the irradiance level of remote phosphors. In contrast to phosphors, no light conversion is required as the diffuser relies solely on strong broadband (full visible range) lossless multiple light scattering events, enabled by a highly porous (>99.99%) non-absorbing nanoarchitecture, resulting in efficiencies of ~98%. This can unleash the potential of lasers for high-brightness lighting applications, such as automotive headlights, projection technology or lighting for large spaces.

## Abstract (web summary)

Laser-based lighting typically relies on wavelength conversion materials that reduce efficiency. The authors present a 3D boron nitride hollow-tube based foam structure that acts as a broadband diffuser with engineered disorder for conversionless white light generation from laser-diode light sources

## Introduction

**Fig. 1:**
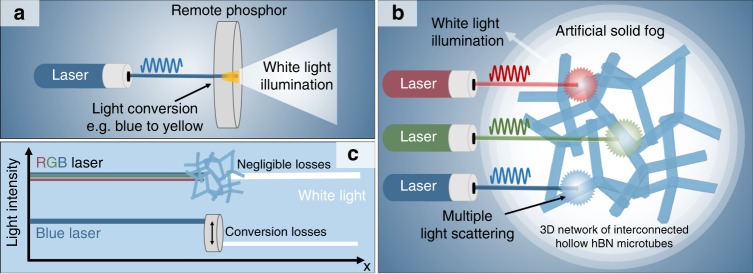
Schematics of laser-based lighting concepts. **a** White-light generation by employing a remote phosphor that converts a part of the blue laser light into yellow light resulting in white light. **b** White-light generation based on an artificial solid fog in combination with an R+G+B laser system. A macroscopically expanded porous (>99.99%) network of interconnected and hollow hBN microtubes with nanoscopic wall thickness is used to convert directed laser light into an isotropic high-brightness white light  source, exploiting multiple light scattering. **c** Schematic comparison of efficiencies of both systems. In the case of remote phosphor, light conversion results in a strong efficiency reduction, whereas the negligible absorption and conversionless light-scattering properties of the hBN foam allow for almost zero losses in light intensity.

Solid-state lighting (SSL) is defined as light emitted by solid-state electroluminescence^1^. Its current power efficiency, i.e., the optical output power of the SSL device per unit input electrical power^2^, is ~70% and there is no fundamental physical reason why efficiencies well beyond 70% could not be reached^2–4^. SSL is thus expected to replace all conventional light sources by 2035^5^, including halogen, xenon, incandescent, and fluorescent lamps^4,6–8^. At present, light emitting diodes (LEDs) are the most efficient devices for white-light generation^2,3,6^. Their adoption is predicted to achieve a 75% reduction of energy consumption for lighting by 2035^5^ in the US alone, which would result in a total energy saving of 6.75 × 10^16^ TJ (equivalent to nearly $630 billion in avoided energy costs) and thus drastically reduce greenhouse emission worldwide^5^. However, the so-called “efficiency droop” still limits the operation of LEDs to very low input power densities, with current densities ~0.01 kA cm^−2^ ^2,9^. Consequently, for a higher light output the physical size of a LED has to be increased. In contrast to LEDs, laser diodes (LDs) can be operated at much higher current densities (>10 kA cm^−2^), with peak efficiencies close to that of LEDs^2^. This results in a higher light output per unit area, e.g., a 0.1 mm^2^ LD source can produce the same amount of light as a 1 cm^2^ LED. Hence, the target to generate more photons at high-power densities (kW cm^−2^) and decrease the cost per lumen can only be satisfied by using LDs^2,4,8^. State-of-the-art LD-based lighting devices exploit a blue LD pumping, e.g., a yellow-light emitting phosphor, resulting in white light (Fig. 1a)^2,4^. However, the performance of such systems is strongly limited by the properties of the phosphor. The efficiency of state-of-the-art light emitting phosphors, such as doped yttrium-aluminum-garnet, is mainly determined by two types of energy losses, the stokes shift (~80% efficiency) and the photoluminescence quantum yield (~90%)^10^. Both these loss mechanisms scale with temperature^10^ (e.g., as a result of illumination) and therefore phosphor luminescence suffers from saturation^10^, aging^11^, and thermal quenching^10^, limiting the irradiance to ~5 kW cm^−2^ and thus the overall light output. Even though new concepts such as glass encapsulation^12,13^, phosphor monoliths^14^, or composite ceramic phosphors^15–17^ can increase the irradiance level up to ~10–20 kW cm^−2^, the true potential of lasers for high-brightness lighting applications, with possible light outputs of several MW cm^−2^, still remains unemployed.

Here, we demonstrate a tunable, disordered, cubic centimetre-sized ceramic nanoarchitecture as an efficient (> 98%) broadband (> 450–640 nm) diffuser, that in combination with a RGB (red-green-blue) laser system (Fig. 1b), is an alternative to the conventional used phosphors with a single laser (Fig. 1a). The diffuser withstands ~10 times the irradiance level achievable by state-of-the-art phosphors, enabling a lighting system whose efficiency is mainly determined by that of the LDs used, due to the lack of any conversion effects (Fig. 1c). The concept is based on a highly porous (> 99.99), macroscopic, and translucent network of randomly arranged and interconnected hexagonal boron nitride (hBN) hollow microtubes, that we call Aero-BN. The material acts like an artificial solid fog, but with a defined hierarchical internal structure - a combination of well separated feature sizes greater than, equal to, as well as below the magnitude of the impinging wavelength. The Aero-BN diffuser enables an isotropic light distribution from a multitude of coherent laser light sources at the same time, while simultaneously reducing speckle contrast to values well below the detection limit of the human eye (< 4%)^18^. Especially the latter is a strict requirement for LD-based lighting, that is not met by today’s commercially available diffuser systems (Supplementary Note 1 and Supplementary Table 1). Even though the current state of LD technology - with laser efficiencies < 20% for green^19^ and < 40% for blue^2,19^ - is still limiting the application of LD-based lighting systems, fast progress in the development of more efficient laser diodes is expected in the near future^2–4,8^. Therefore, the development of new optical components, such as the Aero-BN discussed here, is a necessity, indicating a way to unlock the full potential of LDs for high-brightness illumination, such as needed in projector technology, automotive headlights, large room illumination, and sports lighting.

## Results

### Light diffuser based on interconnected hBN microtubes

**Fig. 2:**
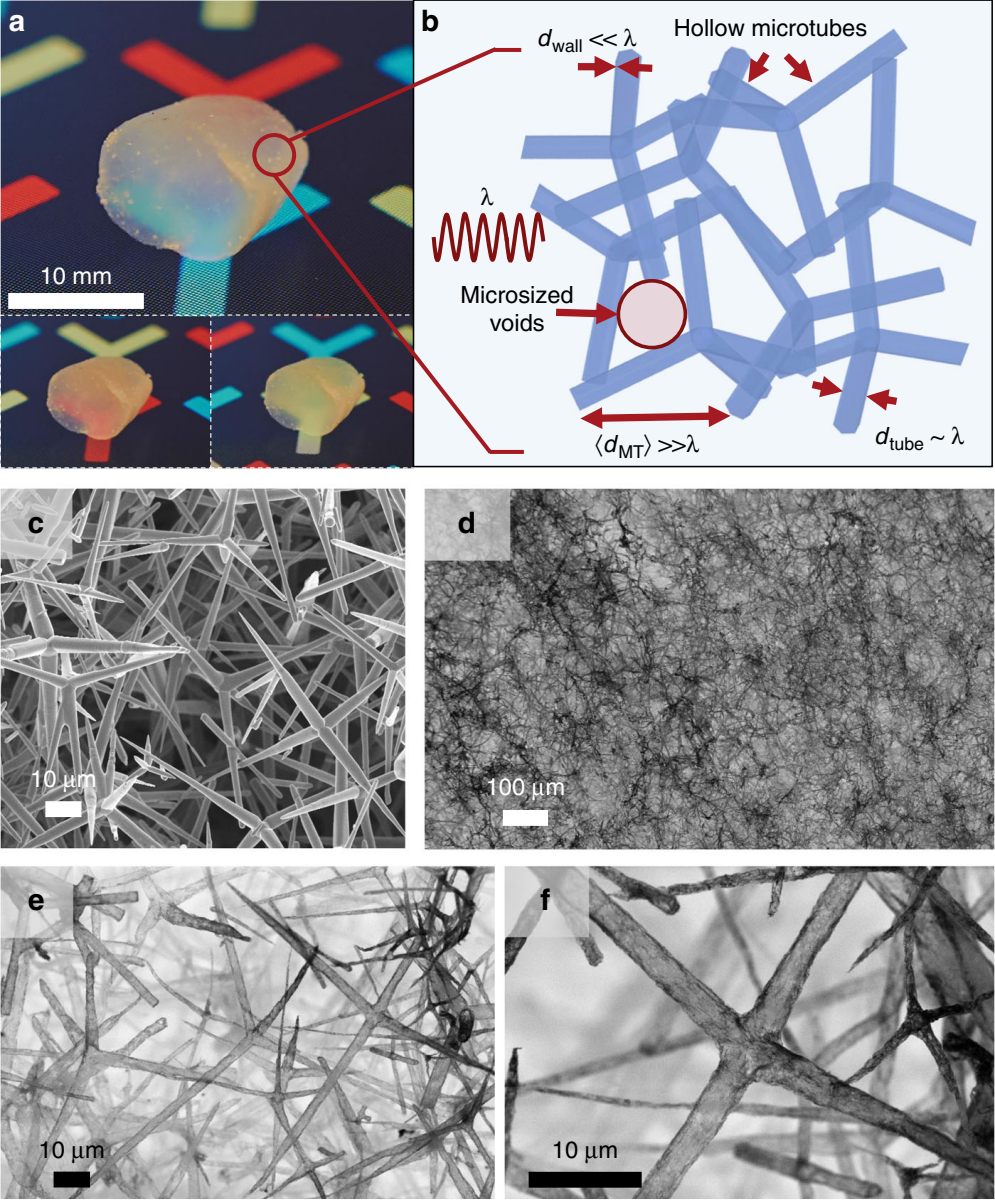
Artificial solid fog. **a** Photographs of Aero-BN.  A thin (< 25 nm) hBN layer is grown by CVD using macroscopically expanded templates of tetrapodal ZnO microparticles. The hBN layer encloses the entire template structure, while it is simultaneously removed by hydrogen etching, resulting in a free-standing, low density (< 1 mg cm^−3^) network, consisting of interconnected hollow hBN microtubes. **b** The structure resembles an artificial solid fog, i.e., a highly optically disordered (completely randomised) photonic system with a combination of feature sizes greater than, equal to, or well below the impinging light wavelength. **c** Representative scanning electron microscopy (SEM) micrographs of the ZnO template consisting of interconnected microrods. **d**–**f** SEM micrographs of the resulting Aero-BN after CVD. The microtubes have an average length ~25 μm, diameter between 300 and 3000 nm, and < 25 nm wall thickness.

The laser light diffuser is based on a macroscopically (> mm^3^) expanded nanoarchitecture consisting of interconnected nanoscopic hBN films (thickness < 25 nm) in the form of hollow tubes, see Fig. 2. hBN has a large band gap of up to 6.5 eV^20^, ensuring low (< 1%) absorption coefficients in the visible light regime. Optical transmission up to 99% at 250–900 nm was reported for thin (1–2 nm) hBN films^21^. Our synthesis process (Supplementary Fig. 1) is based on a ceramic template material (Supplementary Fig. 2)^22^, which offers, in contrast to the common Ni templates^23^ used for the synthesis of hBN and graphene foams, fabrication flexibility, as the template can be tailored^22^ in its density, microstructure (e.g., pore size and pore interconnectivity) as well as geometry. It consists of randomly distributed, interconnected ZnO microrods, with large (up to 100 μm) voids and porosities up to 98%^22^. The synthesis of the final BN network involves a one-step transformation of the ZnO microrod structure in which a thin (< 25 nm) hBN layer is formed by a chemical vapor deposition (CVD) process, while the ZnO template is simultaneously removed (Supplementary Figs. 3 and Supplementary Note 2). The final semitransparent Aero-BN (porosity > 99.99%) microtube network is shown in Fig. 2a. Calculations indicate that the specific surface area of the hBN foams is in the order of 900 m^2^ g^−1^ (see Supplementary Note 3). Energy dispersive X-ray spectroscopy (EDX, Supplementary Fig. 5) show that the ZnO template is completely removed during CVD. The process results in a disordered^24^ macroscopic network, Fig. 2b,  consisting of interconnected hollow hBN microtubes, with individual features varying in well-defined sizes and dimensions. The as-synthesized hollow hBN microtubes have an average length ~25 µm, and their diameter, *d*_tube_, is between 300 and 3000 nm, depending on the geometry of the used ZnO microrods (Fig. 2c) as shown in Fig. 2d–f. Thus, *d*_tube_ is of the same order of magnitude as the wavelength of visible light. The hBN CVD process results in wall-thicknesses *d*_wall_ < 25 nm. This is much smaller than the wavelength of visible light, promoting light-matter interactions that are dominated by Rayleigh-type scattering^25^. As for Fig. 2f, the hBN microtube walls consist of randomly arranged, interconnected hBN nanoplates (see also Supplementary Fig. 6). The average distance between the individual microtubes, *d*_MT_, is several μm, larger than the visible light wavelength. The resulting Aero-BN network architecture leads thus to an optical system with microscopic (optical) density fluctuations (volumes filled with air and with hBN microtubes) throughout the macroscopic structure, as indicated in Fig. 2b. The CVD process is similar to that used to prepare macroscopically expanded nano architectures based on interconnected ZnO microrod networks^26–28^, with the main difference that the hBN is grown here by using the sacrificial ceramic template.

**Fig. 3:**
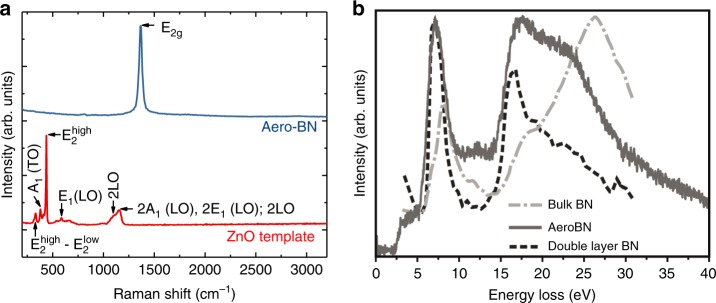
Raman and EELS characterisation of the Aero-BN network. **a** Raman spectra of Aero-BN structure (blue) and ZnO template (red). **b** Low-loss EELS spectra of bulk-hBN^37^ (dash-point), Aero-BN (solid), and double layer hBN^37^ (dashed). The positions and shapes of the π-plasmon at ~6 eV match. The positions of the σ-plasmon ~15 eV match, while shape and relative intensity differ slightly, whereas no peak ~26 eV (bulk-BN) is seen. Spectra are normalized from the onset of the π-plasmon to its apex.

Figure 3a shows Raman spectra (*λ* = 514 nm, 1.32 mW) of Aero-BN (blue curve) and ZnO (red curve). The Aero-BN spectrum has a characteristic single peak centred ~1366 cm^−^^1 ^^29–31^. The ZnO spectrum shows several resonances. The sharp peak ~439 cm^−^^1^ indicates the crystal quality of the sample^32^. The peak at ~335 cm^−1^ is assigned to the difference between $E_2^{{\mathrm{high}}}$ and $E_2^{{\mathrm{low}}}$ [$E_2^{{\mathrm{high}}}$− $E_2^{{\mathrm{low}}}$], which corresponds to the high and low longitudinal optical branches of ZnO, while the feature at 384 cm^−1^ is assigned to A_1_(TO) mode^33^. In addition, the black curve shows a peak at ~584 cm^−^^1^ attributed to E_1_(LO) mode. The broad, intense peak at 1158 cm^−^^1^, which is found between the doubled frequencies measured for the A_1_(LO) and E_1_(LO) modes, contains contributions of 2A_1_(LO) and 2E_1_(LO) modes at the Γ point of the Brillouin zone, and possibly also of 2LO scattering^33^. The weaker peak ~1105 cm^−1^ can be attributed to 2LO at H and K point of the Brillouin zone^33^. However, no peaks of ZnO are observed in the Aero-BN spectrum, consistent with the removal of the sacrificial ZnO template.

Transmission electron microscopy (TEM) investigations reveal, that the atomic structure of Aero-BN resembles that of hBN nanotubes (see Supplementary Note 4 and Supplementary Fig. 7)^34^. Furthermore, high-resolution (HR) micrographs show the existence of numerous point and triangle defects, potentially advantageous for catalytic applications^35^ (Supplementary Fig. 8). The wall thickness of the BN microtubes is determined via the electron energy-loss spectroscopy (EELS) log-ratio method^36^ (Supplementary Fig. 9 and discussion) to be 4–25 nm. The EEL spectra in the plasmon region up to 40 eV are shown in Fig. 3b and compared with those of double hBN layer^37^. The positions of the π-plasmon at 6 eV and the σ-plasmon at 15 eV match the double hBN layer reference ^37^. This confirms the nanoscale thickness of Aero-BN microtube walls, as bulk hBN shows σ-plasmon resonance peaking ~26 eV^37^.

Optical absorption measurements with an integrating sphere (Supplementary Fig. 10) performed on a macroscopic Aero-BN sample (*ρ*_Aero-BN_ ~ 0.68 mg cm^−3^) give absorption ~4.04, 0.85, and 0.11% for blue (450 nm), green (520 nm), and red (638 nm) laser lights, respectively. The slightly larger absorption at 450 nm might be caused by traces of ZnO, however the amount is too low to affect the measurements critically, as the measured absorption is consistent with that of 1–2 nm thick hBN structures^21^. The  low absorbtion in combination with the structural feature sizes greater than, equal to, as well as below the magnitude of the impinging wavelength, results in a disordered system^24^, in which the light transport properties are determined by multiple light scattering.

### Light-scattering characteristics

In order to analyze the light-scattering properties and to determine the underlying mechanisms we fabricate Aero-BN with different densities *ρ*_Aero-BN_ (0.17–0.68 mg cm^−3^) by changing the initial template density *ρ*_T_ between 0.3 and 1.2 g cm^−3^ (see Supplementary Fig. 11). This enables us to tune and control the internal light-scattering properties, key to build the envisaged laser light diffuser. For example, a template density *ρ*_T_ ~300 mg cm^−3^ results in *ρ*_Aero-BN_ as low as ~0.17 mg cm^−3^ (equal to a porosity > 99.99%), lower than that of other reported macroscopically expanded BN architectures^34,38–45^ (see Supplementary Table 2).

**Fig. 4:**
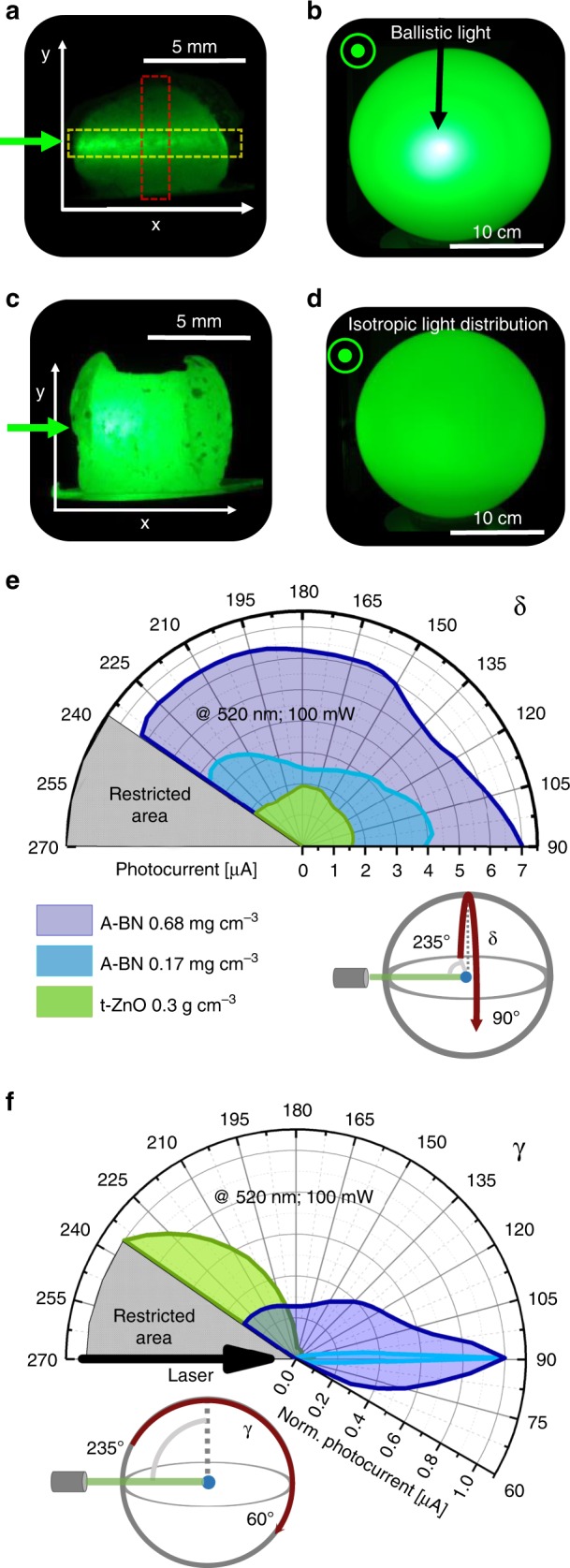
Light-scattering characterisation. **a** Photograph of *ρ*_Aero-BN_ ~0.17 mg cm^−3^ illuminated with 100 mW (spot size ~1 mm) at 520 nm. **b** Photograph of the same sample shown in **a** when illuminated in the centre of a semitransparent glass bulb (front view). **c**, **d** Photograph of *ρ*_Aero-BN_ ~0.68 mg cm^−3^ illuminated with 100 mW at 520 nm, and resultant light scattering imaged using a semitransparent glass bulb (front view). **e** Angular photocurrent dependence for Aero-BN with different *ρ*_Aero-BN_ compared with an interconnected microrod structure (t-ZnO; *ρ*_T_ ~300 mg cm^−3^) for 520 nm at 100 mW. The photodiode is polar rotated over the sample, as illustrated in the schematics. The graphs represent the photocurrent produced by scattered light only. **f** Corresponding normalised photocurrent with respect to the azimuthal rotation of the photodiode. For details of measurements see Supplementary Figs. 17–20.

The light-scattering properties are demonstrated by illuminating an Aero-BN sample from one side with a focused laser. Figure 4a shows a photograph (perpendicular to the laser axis) of a low density (*ρ*_Aero-BN_ of ~0.17 mg cm^−3^) Aero-BN illuminated at 520 nm. The resultant frontal photograph of the same sample illuminated in the centre of a semitransparent glass bulb, Fig. 4b, shows that most of the incident laser beam is transmitted through the material. Figure 4c, d display the corresponding photographs of a sample with a higher initial *ρ*_Aero-BN_ ~0.68 mg cm^−3,^ for 520 nm illumination at 100 mW. As shown in Fig. 4d, a nearly homogeneous, isotropic light distribution, with no visible transmitted primary beam, is seen at the semitransparent glass bulb screen. The corresponding intensity plots, Supplementary Fig. 12, obtained from Fig. 4a (highlighted areas) indicate that the intensity of the incident laser beam decreases linearly through the sample along *x* and *y *directions. This can be described by a system in which the scattering mean free-path l^*^ is much larger than the sample dimensions^46^, resulting in an overall low scattering efficiency (most light is transmitted rather than scattered), i.e., ratio of scattered to transmitted light, and a dominating ballistic transmission. For the ZnO microrod template used to prepare our Aero-BN, with the same microstructure (microrods instead of hollow microtubes), a pronounced visible backscattering is observed (Supplementary Figs. 13–15), indicating the fundamental role of the hollow tubular geometry with multiple feature sizes. A more detailed discussion on the influence of different ceramic microstructural arrangements can be found in Supplementary Note 5 and Supplementary Fig. 16.

The detailed light distribution produced by illuminating Aero-BN samples is investigated with a photo-goniometer^47^ (a photodiode movable around the illuminated specimen on a spherical surface, see Supplementary Fig. 17) to characterise the broadband light-scattering properties as a function of the angle (azimuthal and polar rotation) at 450, 520, and 638 nm, Fig. 4e, f. We also consider a network of interconnected ZnO microrods^48,49^ as a comparison. A perfect 3D light diffuser exhibits angle independent (isotropic) emission over the complete angular range, so that the light is uniformly emitted in all directions. Figure 4e, f show plots of both azimuthal and polar rotations, extracted from the polar plots of the goniometer measurements of the laser illuminated ZnO and Aero-BN networks (see Supplementary Figs. 18–20). In both cases, the photodiode is pivoted, while the sample and the LD are stationary. These graphs provide quantitative data for the amount of scattered, reflected, and transmitted light. Figure 4e depicts the photocurrent for different samples as a function of polar angle. This represents the photocurrent produced by scattered light only, while no reflected and transmitted light reaches the detector. In contrast, Fig. 4f shows the normalised photocurrent as a function of azimuthal angle. In this case, the photocurrent detected for 90° < *γ* < 180° is caused by scattering only. The value at *γ* = 90° represents transmission (*T*) and forward scattering. For *γ* > 180° the photocurrent is a result of reflection and scattering. As depicted in Fig. 4e, the ZnO network with *ρ*_ZnO_ ~300 mg cm^−3^ shows only a small but homogeneous photocurrent (~1.5 µA) caused mainly by back-scattered light. Thus, nearly no light is transmitted through the structure, Fig. 4f. Aero-BN, on the other hand, shows a much stronger emission and more uniform light distribution of the laser beam. The measured photocurrent caused by azimuthally scattered light from the Aero-BN (Fig. 4e) is ~2.5–4 µA and 6–7 µA for *ρ*_Aero-BN_ ~0.17 and ~0.68 mg cm^−3^, respectively. Thus, for the higher density sample, the amount of scattered light is ~4.6 times higher than for the ZnO network, even though the density of the network structure is reduced by a factor of ~440. As illustrated in Fig. 4f, the amount of light transmitted (*γ* = 90°) through *ρ*_Aero-BN_ ~0.68 mg cm^−3^ is ~4 times higher than the reflected (and scattered) light (*γ* > 180°). The ratio between transmitted and scattered light (ideal value of 1 for an isotropic diffuser) decreases with increasing network density. For *ρ*_Aero-BN_ ~0.17 mg cm^−3^ this is ~200, whereas it is ~3.7 for *ρ*_Aero-BN_ ~0.68 mg cm^−3^. A value of 1 might be achieved by increasing *ρ*_Aero-BN_ further. The average scattering intensity *S* is calculated by averaging the photocurrent intensities of the polar plots for 105° < *γ* < 170° and 100° < *δ* < 230°. The relative deviation of *S* with respect to *T* (*γ* = 90°) is illustrated in Supplementary Fig. 21 as a function of the optical areal density, i.e., the density times the sample length, for three wavelengths. By increasing the optical areal density (*ρ*_Aero-BN_ × *L*), the ratio (*T-S*)*/S* by over three orders of magnitude, irrespective of the wavelength. Light with a shorter wavelength is scattered more effectively, as for Rayleigh scattering^50,51^ (see Supplementary Fig. 22). Further details on the light-scattering properties are in Supplementary Note 6 and Supplementary Figs. 23–27, showing that the multiple light scattering observed in Aero-BN is a result of the combination of negligible absorption losses and a control of density of scattering centres over several orders of magnitude. Furthermore, we show independent tunability of the density in all three dimensions (given the almost null equivalent Poisson’s ratio of such low-density foam materials^52^, see also Supplementary Fig. 24). This enables control of light diffusion and a nearly constant density of photons close the surface, with at most a linear decay in one dimension.

### Speckle contrast reduction

**Fig. 5:**
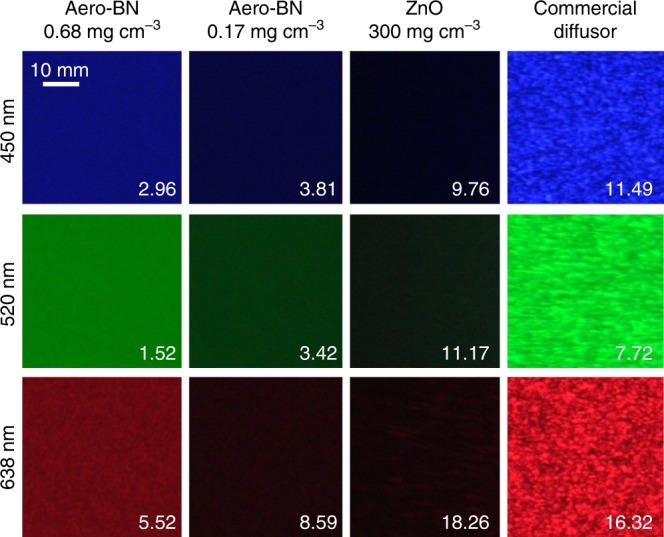
Speckle contrast reduction. Objective speckle pattern at 450, 520, and 638 nm for two Aero-BN samples with high (*ρ*_Aero-BN_ ~0.68 mg cm^−3^) and low (*ρ*_Aero-BN_ ~0.17 mg cm^−3^) density, a porous ZnO microrod network (*ρ*_*T*_ ~300 mg cm^−3^) and a commercial plate diffuser. Values for speckle contrast are in %.

The scattering behaviour also enables us to use Aero-BN for laser illumination without recognizable speckle patterns, thus solving one of the main challenges of using LDs as a light source^18,53,54^. Speckle is the result of interference of light beams with the same frequency, but different phase and amplitude, resulting in a wave with random amplitude variations^55^. The most promising approach to avoid speckle is to use an optical downstream component that superimposes multiple speckle patterns at once^18,53,56^, so that on average no pattern is visible to the human eye, for an exposure time ~1/60 s^57^. In our Aero-BN, the primary laser beam is scattered multiple times. Thereby it is split into a large number of independent beams, causing multiple overlapping speckle patterns. This reduces the objective speckle contrast *χ* (i.e., the mean intensity of the speckle pattern divided by the standard deviation of the intensity) down to ~2%, lower than that for the human eye (4%)^18^. Figure 5 plots the objective speckle pattern for different wavelengths as a function of material and Aero-BN density (*ρ*_Aero-BN_). For high-density Aero-BN (*ρ*_Aero-BN_ ~0.68 mg cm^−3^) the speckle contrast is lowest, with minimal values ~2.96%, 1.52%, and 5.52% for 450, 520, and 638 nm (each at 100 mW), respectively. Therefore, nearly no speckle can be observed by the human eye. Even lower speckle contrast could be achieved by using higher *ρ*_Aero-BN._ Our Aero-BN outperforms commercially available plate diffusers like DG10-220 (*Thorlabs*) in terms of speckle contrast, since these have >5 times higher speckle contrasts (16%). It also surpasses that of the interconnected ZnO microrod networks, as no pure light diffusion can be reached there (see also Supplementary Fig. 15), due to missing Rayleigh-type scattering centres at the nanoscale. The lower speckle contrast for Aero-BN at lower wavelengths is a direct effect of the wavelength dependence (*λ*^−4^) of Rayleigh scattering^50,51^. Due to continuous beam splitting by multiple light-scattering processes, the low speckle contrast might also be related to small (< 50 nm) thermally activated movements of the hollow microtubes with wall thicknesses < 25 nm, resulting in a time-varying speckle pattern (see also Supplementary Note 7 and Supplementary Table 4). This is similar to the speckle contrast reduction obtained by using colloidal dispersions, with values as low as 3% due to particles Brownian motion^18^. In comparison with other methods to reduce speckle contrast, e.g., by random lasing^58^, using small moving diffusers^53^, rotating ground glass diffusers^59^ or nonmoving Hadamard matrix diffusers^60^ our approach does not require complex micromechanical devices, making it easier to use and less prone to failure (see also Supplementary Note 1 and Supplementary Table 1).

### Tunable RGB laser light illumination

**Fig. 6:**
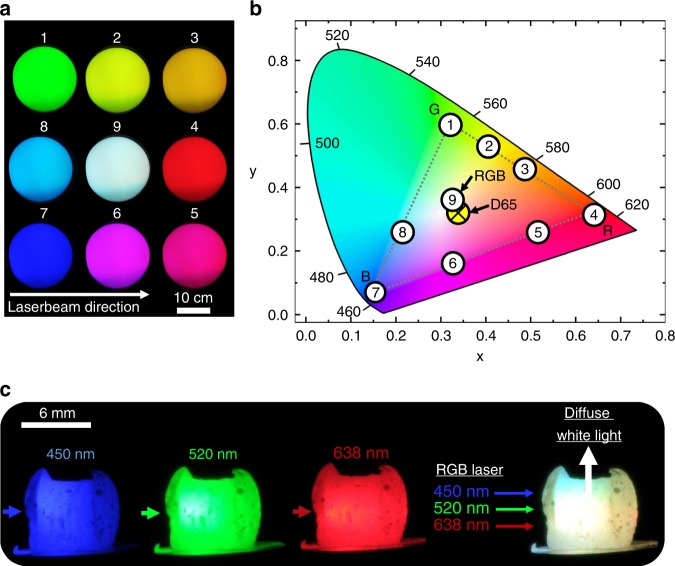
Colour mixing. **a** Light distribution of a high *ρ*_Aero-BN_ sample illuminated in the middle of a translucent glass bulb under different intensities for the each wavelength (450, 520, and 638 nm). White light is produced when all lasers (R + G + B) are at the maximum power (100 mW). **b** CIE colour map with marked values for the pictures in **a**. The value obtained for mixing R + G + B is close to the CIE standard white illuminant D65^62^ (yellow circle). **c** Photographs of a sample with *ρ*_Aero-BN_ ~0.68 mg cm^−3^ illuminated at 450, 520, and 638 nm (100 mW each, 1 mm spot size), respectively, as well as the resultant white light produced if all lasers are used at once. The arrows mark the direction of the incident laser beam.

The viability of our Aero-BN in combination with an RGB laser system as an illumination source, as an alternative to remote phosphors, is demonstrated by illuminating the *ρ*_Aero-BN_ ~ 0.68 mg cm^−3^ sample at different laser intensities under a translucent glass sphere screen. The resulting images are presented in Fig. 6a together with the respective International Commission on Illumination (CIE) colour space values marked in the colour map of Fig. 6b. An all-primary RGB laser wavelength mixing approach, i.e., a combination of three (red, green, and blue) or even four (red, yellow, green, and blue) laser wavelengths is known to outperform the efficiency of any other known white-light source^2–4,8^. Furthermore, the possible colour gamut (i.e., the subset of colours which can be accurately represented) of such a system is on par to that of LEDs or LCDs^61^. By tuning the individual intensities of our RGB laser source, all colours in the resultant RGB triangle (Fig. 6b) can be produced. For the maximum intensity of all lasers, white light is produced, close to the CIE standard white illuminant D65^62^. The corresponding photographs of *ρ*_Aero-BN_  ~0.68 mg cm^−3^ illuminated at 450, 520, and 638 nm are in Fig. 6c, together with a photograph of the same sample illuminated with all wavelengths at once, resulting in a diffuse white-light illumination. Thus, our Aero-BN is an ideal broadband diffuser (see also Supplementary Movie 1) and can be used to fabricate tunable RGB laser light sources with a large colour gamut, depending only on characteristics of the actual laser system used, rather than on light conversion effects such as in the case of remote phosphors.

### Laser damage threshold

To demonstrate that Aero-BN can overcome the irradiance levels of state-of-the-art phosphors needed for high-brightness illumination applications, such as automotive headlights or projectors, we characterised its thermal decomposition and laser damage threshold. Thermogravimetric analysis (TGA) under nitrogen atmosphere indicates nearly no change in weight (±2 wt% up to 1000 °C). In an oxygen-containing atmosphere (nitrogen/oxygen ~1/4) the material is stable up to 700 °C, where the formation of B_2_O_3_ starts^63^ (Supplementary Fig. 28). The chemical reaction also confirms the presence of hBN over other crystalline forms of BN such as wurtzite boron nitride (wBN) and cubic boron nitride (cBN)^64^. To determine the laser damage threshold we use a focused (spot diameter ~8.4 µm) high-power (3 W) continuous wave laser at 450 nm. The threshold is determined by moving the focused laser beam over an individual tube and simultaneously recording the microtube with a charge-coupled device (CCD) camera. After each passage, the laser power is increased until the laser induces morphological damage (e.g., microtube destruction, see Supplementary Fig. 29). However, even at the highest power (~650 kW cm^−2^) the Aero-BN network remains intact, whereas a commercially available state-of-the-art phosphor shows degradation at ~80 kW cm^−2^ (see Supplementary Fig. 30). In contrast to Aero-BN, the phosphor actively converts the incident laser light into energy, which leads to increased heat accumulation. To achieve even higher power densities we use a highly focused pulsed laser (spot diameter ~1 µm) at 355 nm, with 100-Hz repetition rate ~7-ns pulse duration (see Supplementary Fig. 29). In this configuration the Aero-BN shows a high laser damage threshold ~430 MW cm^−2^ (~2.65 J cm^−2^), ~10 times higher than commercially available phosphor (see Supplementary Fig. 31)^12,13,17^. This is directly related to the microscopic structure of the Aero-BN. The nanoscopic wall thickness leads to high transmittance of the individual tubes, meaning that only a small portion of the laser light interacts with a single tube. Furthermore, the low hBN absorption in the visible spectrum^21^, implies that a minimal amount of energy is transformed into heat. The high heat conductivity (~400 W m^−1^ K^−1^)^23^ of hBN helps to quickly transport thermal energy away from the illuminated spot^65^. The high porosity (> 99.99%), the small wall thickness < 25 nm, as well as the micrometre-sized voids enable efficient heat management, similar to that reported for other foam-like nanostructures, such graphene foams^66,67^, since heat can be easily transported to the surrounding air. Furthermore, the volumetric heat capacity of our Aero-BN foam is comparable with that of the surrounding air, as both have similar densities ($\rho _{{\mathrm{air}}}\sim 1.2\,{\mathrm{mg}}\,{\mathrm{cm}}^{ - 3};0.17\,{\mathrm{mg}}\,{\mathrm{cm}}^{ - 3}\,<\,\rho _{{\mathrm{Aero}} - {\mathrm{BN}}}\,<\,1\,{\mathrm{mg}}\,{\mathrm{cm}}^{ - 3}$). Therefore, the damage threshold is only an estimate for the lower destruction limit. The macroscopic destruction threshold of Aero-BN is potentially much higher when a macroscopic laser beam is used, not focused to such a small spot.

## Discussion

We demonstrated a concept for high-brightness and broadband laser illumination based on a diffuser consisting of a network of interconnected hollow hBN microtubes, overcoming the problems associated with inorganic phosphor materials. Their structurally disordered arrangement, combined with the nanoscopic wall thickness, and low absorption are key to enable homogeneous light diffusion through the cm^3^-sized networks and promote light-scattering properties suitable for laser illumination applications. Our Aero-BN has efficient Rayleigh-type scattering centres arranged in thinly spread and controlled manner, resulting in non-exponential light diffusion. By controlling the density of the aero-material system, we are able to adjust the light diffusion so that multiple scattering events result in an almost homogeneous, isotropic light illumination.

For Aero-BN densities ~0.68 mg cm^−3^ the speckle contrast is well below the perception threshold of the human eye. The highly porous structure, together with the low absorption in the visible range, as well as the low volumetric heat capacity and high heat conductivity, enable an efficient heat management. We achieve laser irradiance levels ~10 times higher than commercially available remote phosphors, unleashing the full potential of laser diodes for high-brightness illumination. Being based on multiple light scattering, rather than on light conversion effects, the broadband properties of our diffuser enable an all-primary RGB laser approach for white-light generation and full-colour range mixing with a large colour gamut^8^, without efficiency reduction, thereby overcoming the problems associated with state-of-the-art remote phosphors (see also Supplementary Note 8). With the expected increase in LD efficiencies in the near future,  our concept paves the way to design a new generation of highly effective light sources.

## Methods

### Fabrication of highly porous ZnO networks

The t-ZnO ceramic networks are produced by a flame transport synthesis technique^68^. Zinc powder with a grain size ~1–10 µm is mixed with polyvinyl butyral in a mass ratio of 1:2. The mixture is then heated in a muffle furnace at 60 °C min^−1^ to 900 °C for 30 min. After that a loose powder of ZnO tetrapods is obtained, then pressed into pellets (e.g., *height* ~10 mm, *diameter* ~12 mm) with a density ~0.3 g cm^−3^. Reheating the pellets for 5 h at 1150 °C leads to junctions between the tetrapods and an interconnected network.

### Fabrication of Aero-BN

In Supplementary Fig. 3 the computer-controlled CVD setup for the fabrication of the Aero-BN is illustrated. The highly porous (up to 98%) ZnO ceramic template is placed in the middle of a quartz tube furnace in a ceramic crucible. Next to that (~1 cm), a crucible filled with B_2_O_3_ is placed into the furnace. The reactor is flushed with Ar and the pressure adjusted to 30 mbar. The Ar flow is then adjusted to 30 sccm and the temperature is increased to 910 °C (heating rate ~20 °C min^−1^). Urea is used as a nitrogen source, located in an evaporator which is connected to the quartz tube furnace as illustrated in Supplementary Fig. 3. When the quartz tube furnace reaches 910 °C the evaporator for urea is switched on. By heating to 170 °C at 30 mbar NH_3_ forms^69^, which decomposes to N and H_2_ in the reaction zone of the reactor^70^. At the process temperature (910 °C) N and B react at the surface of the ceramic template, forming a thin (<25 nm) hBN layer. Simultaneously the ZnO template is etched by hydrogen. After 3 h the urea evaporator and the quartz tube furnace are switched off. When the reactor reaches 30 °C the Ar flow is switched off and the sample is removed. A detailed discussion of the reaction is in Supplementary Note 2.

### Characterisation

The morphologies of the different structures are investigated by SEM (Zeiss Supra 55VP) equipped with an EDX detector. Aero-BN is analysed by a FEI Tecnai F30 G2 STwin TEM (300 kV acceleration voltage, cs-coefficient 1.15 mm) and a FEI Titan G2 60-300 TEM equipped with a monochromator. Macroscopic aggregates of Aero-BN are tapped with TEM grids in order to transfer some tetrapods or single fragments onto the grid, minimising the breaking rate for the Aero-BN network. Unfolded BN sheets are also analysed by HRTEM to visualise atomic scale defects. The electronic structure is investigated by HR-EELS with a GIF Quantum/Enfina energy analyser. TGA measurements are performed using a TA Instruments Q50 under nitrogen and nitrogen/oxygen (1/4) at a scan rate of 10 °C min^−1^ from 25 to 1000 °C. Raman spectroscopy is done with a Renishaw 1000 InVia micro-spectrometre at 514.5 nm for the ZnO template and a Witec Instruments Alpha300 RA at 532 nm for the Aero-BN sample.

### Reflectivity calculations

Reflectivity calculations as a function of wall thickness for a hollow hBN microtube and ZnO microrods as a function of diameter for different wavelengths, respectively, follow those in ref. ^71^. Refractive indexes of 1.8^20^ and 2.1^72^ are used for hBN and ZnO. The mean reflectivity is derived by averaging that for incident beam angles of 0–180° (step size of 1°). For each angle the unpolarised and polarised reflectivity is derived. This procedure is repeated for different hBN wall thicknesses as well as ZnO microrod diameters.

### Light-scattering measurements

A photodiode (FDS1010, Thorlabs) is rotated around the sample with an angular step ~5° at a distance ~15 cm, using a photogoniometer. From one side the cylindrical samples are illuminated with an RGB laser (RTI OEM 300 mW RGB Modul, LaserWorld). The sample is positioned so that the laser beam illuminates it in the middle. The spot size is adjusted by a lens to ~1 mm. Each laser has a maximum output power of ~100 mW.

### Absorption measurements

Absorption measurements are performed using an integrating sphere (Opsytec) with an inner diameter of 200 mm, coated with a reflective BaSO_4_ thin film. The illumination intensity is measured by connecting to it a radiometer (RM-22, Opsytec). The sample is mounted on a thin (diameter of 3 mm) Al slab in the centre of the sphere. Through an opening of 2 mm, the laser is focused on the sample. The absorption is calculated as the ratio of the luminous flux measured by the radiometer with and without sample. This is integrated for at least 20 s.

### Transmission measurements

Transmission measurements are performed using the same integrating sphere used for absorption. The sample is placed in front of a 2 mm opening of the sphere. The laser is adjusted to be in the same axis as the opening of the sphere and focused on the sample. The transmission is calculated as the ratio of the measured luminous flux with and without sample. For measurements as a function of compression, the sample is clamped between two highly reflective (>99%) plates to ensure as little light absorption as possible by the surrounding (clamping) material. The sample is compressed step by step using a high-precision screw. After each compression, a transmission measurement is performed as described before, using an integration time of at least 20 s. This is increased to 60 s for small fluxes.

### Laser damage threshold

The sample is moved using a *xy*-translation stage, such that the laser beam directly hits an individual nanostructure, e.g. a microtube. The laser focus is adjusted using the back-scattered signal of the laser spot, tuned towards its highest intensity by moving the translation stage in *z*-direction. The laser signal is then filtered on a video camera by using a notch filter, while only the microscope image is monitored. The laser power is increased stepwise until the first morphological changes of the nanostructures become evident in the microscope (white light) image. The corresponding laser intensity (on the sample) defines the destruction threshold of the investigated materials. The commercially used phosphor is a Intematix CL830R45XT.

### Speckle pattern photography and contrast

Objective speckle patterns (i.e. the intensity pattern produced by the interference of a set of wavefronts) are obtained by illuminating with a focused laser beam with 100 mW at 450, 520, and 638 nm. The objective speckle pattern forms on a sheet of white paper at 90° with respect to the incoming laser. The distance between sample and speckle pattern is ~40 cm. The pattern is photographed using a CCD camera (Nikon D300) equipped with a lens with 120 mm focal length. The camera is positioned slightly over the sample, to avoid any light being directly scattered into the lens. The aperture of the lens is used at maximum of f/4 to take as much light in as possible. Since speckle patterns are time dependent^56^, the exposure time is important. We use 1/60 s, close to the detection limit of the human eye^57^. To avoid any overexposure of the CCD chip we use a camera sensitivity (ISO) of 800. For weakly scattering samples, this might lead to a dark speckle pattern. However, this has no influence on the speckle contrast, whereas an overexposure would result in wrong calculations. The photographs are taken at a maximum resolution of 2848 × 4288 pixels. The speckle contrast of the resultant photographs is calculated by using the Gatan Microscopy Suit. A representative quadratic area (several cm^2^) is chosen. The colour is converted into a black and white representation. From these images the mean intensity Φ as well as the standard deviation *σ* is calculated using the above mentioned software. The speckle contrast *χ* is than calculated as follows^56^:

$$\chi\,=\,\frac{\sigma }{\Phi }.$$

### FEM simulations

The FEM model developed to compute the network variation of projected porous areal density and Poissonʼs ratio under monoaxial compression (see Supplementary Note 6), consist of a periodic supercell ~71 × 83 × 46 μm^3^ (*x*, *y*, *z*) containing nine tetrapods mutually interconnected. We consider an average geometry of the tetrapod with *d*_air,1_ = 1.67 µm, *d*_air,2_ = 1.00 µm, *t*_wall_ = 4 nm, and *r* = 27 or 38 µm^73^ to simulate networks with high or low densities, (respectively *ρ*_Aero-BN_ = 0.367 mg cm^−3^ and *ρ*_Aero-BN_ = 0.178 mg cm^−3^), similar to the ones tested in the experiments. Tetrapods are built associating the arm extremities and the central joint of the tetrapods to the vertexes and centroid of a regular tetrahedron, respectively. The tube walls are modelled with thin shell elements with selective-reduced integration, and the spurious modes effects are controlled. Monoaxial compression tests are reproduced with periodic boundary conditions along the lateral faces of the supercell (*x* and *y* directions) while the two horizontal rigid surfaces act to apply the monoaxial load on the network along *z* (displacement controlled, 0.25 μm ms^−1^). Contact between tetrapods and within elements of the same tetrapods are implemented to prevent mutual and self-penetration. The density of the supercell is monitored along the simulations. To measure the evolution of the projected porous area, images of lateral view of the network (*xz* and *yz* planes) are extracted from simulations at a constant time sampling. The normalised projected porous area (Ω/Ω_0_) is measured via a graphics software (paint.net) by selecting the void area in the lateral projection fo the network (“magic wand” tool) and computing the corresponding number of pixels (ratio of the current vs. initial value).

## Supplementary information

Supplementary Information

Description of Additional Supplementary Files

Supplementary Movie 1

## Data Availability

The data that support the findings of this study are available from the corresponding authors upon request.
