# Supplementary material for: Conversionless efficient and broadband laser light diffusers for high brightness illumination applications
Source: Nat Commun. 2020 Mar 18;11:1437. doi: 10.1038/s41467-020-14875-z (PMC7080714; doi:10.1038/s41467-020-14875-z)
Supplement: Supplementary file 1 — Supplementary Information [file 41467_2020_14875_MOESM1_ESM.pdf]

Supplementary Information to:

Conversionless Efficient and Broadband Laser Light Diffusers for High Brightness Illumination Applications

Fabian Schütt[1], Maximilian Zapf[2], Stefano Signetti[3], Julian Strobel[4], Helge Krüger[1], Robert Röder[2], Jürgen Carstensen[1], Niklas Wolff[4], Janik Marx[5], Tian Carey[6], Marleen Schweichel[1], Maik-Ivo Terasa[1], Leonard Siebert[1], Hyo-Ki Hong[8], Sören Kaps[1], Bodo Fiedler[5], Yogendra Kumar Mishra[7], Zonghoon Lee[8,9], Nicola M. Pugno[3,10,11], Lorenz Kienle[4], Andrea C. Ferrari[6], Felice Torrisi[6,12], Carsten Ronning[2], Rainer Adelung[1*]*

[1]*Functional Nanomaterials, Institute for Materials Science, Kiel University, Kaiserstr. 2, 24143 Kiel, Germany*

[2]*Institute for Solid State Physics, Friedrich-Schiller-University Jena, Max-Wien-Platz 1, 07743 Jena, Germany*

[3]*Laboratory of Bio-inspired, Bionic, Nano, Meta Materials & Mechanics, Department of Civil, Environmental and Mechanical Engineering, University of Trento, via Mesiano 77, I-38123 Trento, Italy*

[4]*Synthesis and Real Structure, Institute for Materials Science, Kiel University, Kaiserstr. 2, 24143 Kiel, Germany*

[5]*Institute of Polymers and Composites, Hamburg University of Technology, Denickestr. 15, 21073 Hamburg, Germany*

[6]*Cambridge Graphene Centre, University of Cambridge, 9, JJ Thomson Avenue, Cambridge CB3 0FA, UK*

[7]*SDU NanoSYD, Mads Clausen Institute, University of Southern Denmark, Alsion 2, 6400, Sønderborg, Denmark*

[8]*School of Materials Science and Engineering, Ulsan National Institute of Science and Technology (UNIST), Ulsan 44919, Republic of Korea*

[9]*Center for Multidimensional Carbon Materials, Institute for Basic Science (IBS), Ulsan 44919, Republic of Korea*

[10]*School of Engineering and Materials Science, Queen Mary University of London, Mile End Road E1 4NS, London, United Kingdom*

[11]*Ket-Lab, Edoardo Amaldi Foundation, via del Politecnico snc, I-00133 Roma, Italy*

[12] *Department of Chemistry, Molecular Sciences Research Hub, Imperial College London, White City Campus, Wood Lane, W12 0BZ, United Kingdom*

Corresponding Authors:

Prof. Dr. Rainer Adelung (ra@tf.uni-kiel.de)

Dr. Fabian Schütt (fas@tf.uni-kiel.de)

Supplementary Note 1. Comparison of different laser diffuser systems

The aim of a laser light diffuser is to broaden/diffuse a directed laser beam.[1] Sophisticated diffusers are also required to eliminate, or at least reduce, speckle contrast well below the limit of the human eye.[1] Thereby laser light can be used for practical lighting applications. Furthermore, the diffuser is required to have low absorbance in order not to reduce the efficiency of the lighting system. Almost all standard diffusers that are available on the market today have a plate-like geometry[2] and can be divided into two subclasses depending on their scattering characteristics, i.e. bulk scattering or surface scattering. **Supplementary Table 1** gives an overview of the different types of laser light diffusers, compared to that presented here. Bulk scatters typically rely on porous polymer diffusers[3], e.g. by introducing small air pockets into the polymer during fabrication. However, these systems are strongly limited in terms of their laser damage threshold and also in the efficiency due to light absorbtion. In contrast to that, surface scatters usually consist of a glass substrate that is coated with a very rough, and thus strongly scattering, thin film.[1] The surface of the glass itself can be microstructured, e.g. by pulsed laser patterning to form a strongly scattering thin film[2]. Surface scattering diffusers provide a higher laser damage threshold and also have a higher transmission efficiency[4] (~90%) compared to bulk scattering diffusers. Nevertheless, they are strongly wavelength dependent.[2] The main disadvantage is their low scattering strength, since most of the laser light is only scattered once or twice, resulting in high speckle contrasts (> 10%) unsuitable for light illumination.

To further reduce speckle contrast, different techniques have been employed so far, such as moving[5], vibrating[4] and rotating diffusers[6]. These techniques result in a temporal averaging of the produced speckle pattern so that it is not recognizable by the human eye anymore. However, those system are strongly susceptible to mechanical failure and require additional components, thereby reducing practicability. Other approaches to reduce speckle are based on non-moving Hadamard matrix diffusers[7].

Supplementary Table 1. Comparison of different light diffuser concepts and their characteristics.

Type of Diffuser		Schematic working principle	Scatter Loss	Laser Damage Threshold	Speckle Contrast	Moving Parts
Diffuser based on bulk scattering	Porous Polymer diffuser	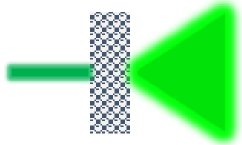	Very High	Very Low	High	No
	Aero-BN diffuser	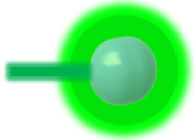	Very Low	Very High	Very low	No
Diffuser based on surface scattering	Ground glass diffuser	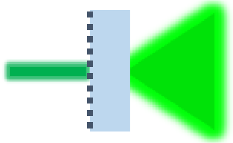	Low	High	High	No
	Vibrating/Moving ground glass diffuser	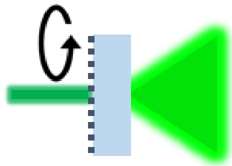	Low	High	Low	Yes

Supplementary Note 2. Fabrication process, synthesis mechanism and additional SEM/EDX characterizations of Aero-BN

Our diffuser is based on a 3D architecture of randomly interconnected hollow hexagonal boron nitride (hBN) microtubes with a wall thickness < 25 nm. This is assembled with a method (**Supplementary Figure 1**) based on a highly porous (up to 98%) ceramic zinc oxide (ZnO) template (**Supplementary Figure 2**). Other methods based on a template approach for the fabrication of BN 3D architectures exist[8–13], however they involve multiple fabrication steps and do not result in a randomly and disordered framework structure consisting of low thickness (< 50 nm) hBN tubes, necessary for our diffuser application (see **Supplementary Table 2**). In **Supplementary Figure 3** the CVD setup for the fabrication of the Aero-BN is illustrated. The highly porous (up to 98%) ZnO ceramic template (see **Supplementary Figure 2**) is placed in the middle of a quartz tube furnace in a ceramic crucible. Next to the template there is a crucible filled with B_2O_3. The reactor is flushed with Ar and the pressure is adjusted to 30 mbar. After that, the Ar flow is adjusted to 30 SCCM and the temperature is increased to 910 °C. At this pressure and temperature B_2O_3 sublimates[14] and reacts with the ZnO template. Representative SEM images of the resultant reaction products are shown in **Supplementary Figures 3b-g**. In contrast to the ZnO template (**Supplementary Figure 2**), the surface morphology changes, consisting of several islands. In the locations where some of the rods are fractured (e.g. during SEM preparation), another phase forms on the ZnO template, with the core of the rods still being ZnO (see **Supplementary Figure 3**). XRD confirms that this phase is related to zinc borate, see **Supplementary Figure 4**. The formation of a thin zinc borate layer on the template by the reaction between the sublimed boron and the ZnO is one key in our synthesis method for Aero-BN. The melting point of zinc borate is ~960 °C.[15] However, at reduced (< 1 atm) pressures the melting

point of most solids is reduced, due to the favorable expansion.[16] Thus, at 910 °C the sublimed B_2O_3 is able to react with the ZnO template forming a homogenous liquid film of zinc borate around the ZnO template. We use urea as a nitrogen source. When the quartz tube furnace reaches 910 °C the evaporator for urea is switched on. By heating urea to 170 °C at 30 mbar NH_3 forms[17], which decomposes to N and H_2 in the reaction zone of the reactor.[18] At the process temperature (910 °C) H_2 etching of ZnO is possible.[19] During the etching of ZnO and zinc borate, N is able to react with the B in the zinc borate phase, forming a thin (< 25 nm) hBN layer (see **Supplementary Figure 6**). A similar reaction mechanism was reported for the fabrication of aerographite tetrapodal networks.[19–21]

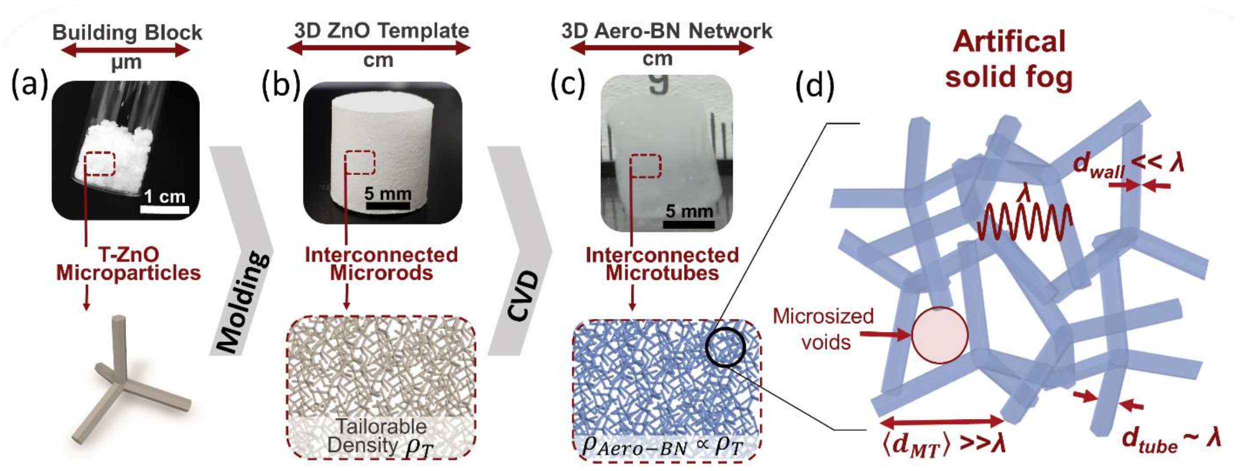

Supplementary Figure 1. Schematic Aero-BN fabrication process: a) Powder of tetrapod shaped ZnO (t-ZnO) microparticles used for the fabrication of the macroscopically expanded networks shown in in b). By adjusting the mass of the tetrapods, as well as the volume of the network mold (up to cm^3 scale) an interconnected microrod network with adjustable density ($\rho_{\mathbf{T}}$) can be fabricated. c) A thin (< 25 nm) hBN layer is then grown by CVD, enclosing the entire template structure, while the template is simultaneously removed by hydrogen etching. The density of the resulting network ($\rho_{\text{Aero-BN}}$) is proportional to ρ_{T}. d) The process results in an artificial solid fog, a highly optically disordered photonic system with feature sizes greater than, equal to or well below the impinging light wavelength, as well as microscopic density fluctuations.

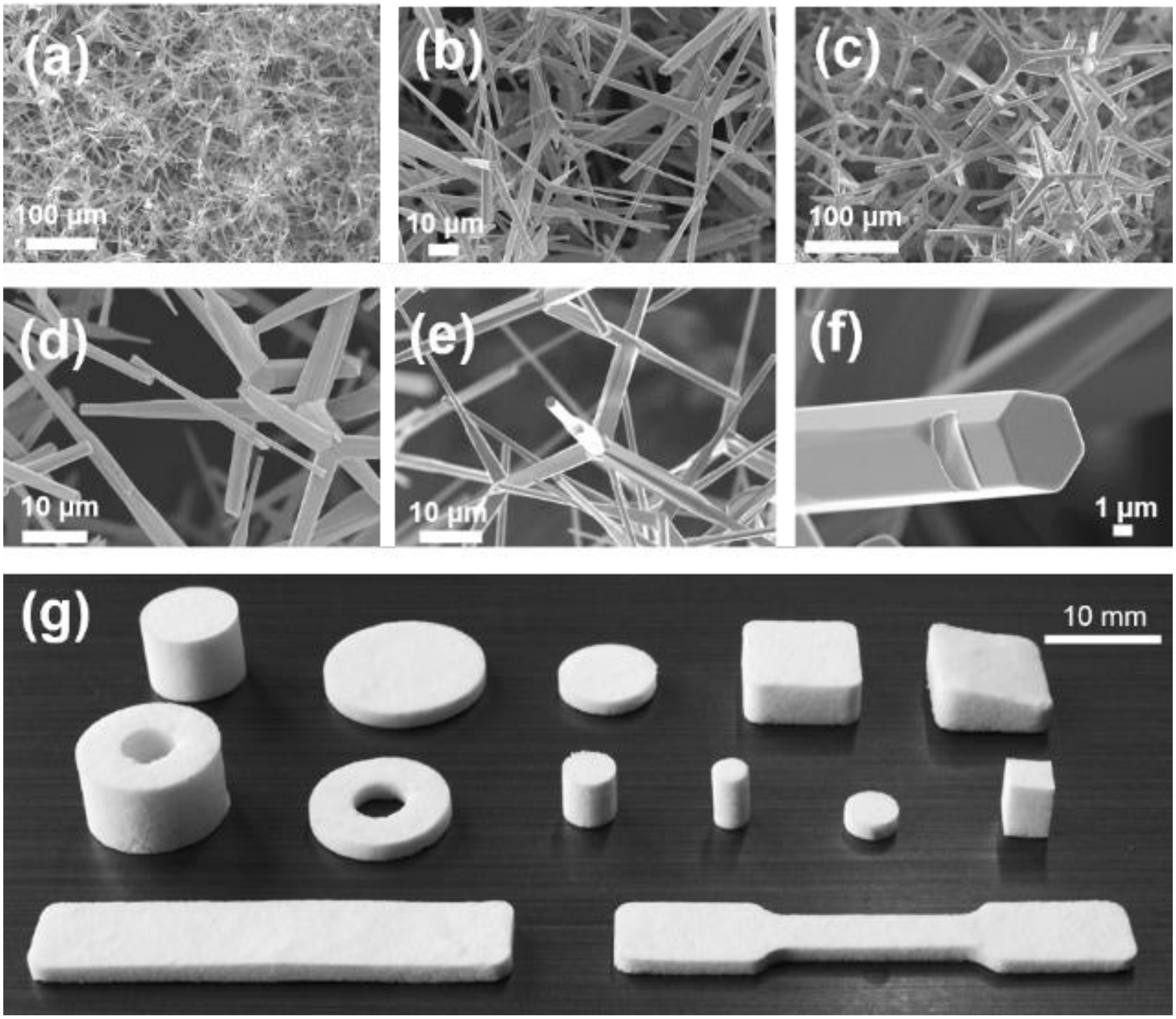

Supplementary Figure 2. t-ZnO template: a)-d) SEM images of the porous template consisting of interconnected tetrapodal shaped ZnO particles, fabricated by flame-transport synthesis. Tetrapods have a mean arm length ~ 25 µm and an arm diameter ~ 0.3 to 3 µm. e) Junctions between the particles formed during sintering. f) High magnification SEM of a single tetrapod arm. g) Examples of macroscopic ZnO template geometries (all with ~ 0.3 g cm^{-3} density) that can be produced by molding.

Supplementary Table 2. 3D hBN architectures in literature and their corresponding densities.

3D hBN	Reference	Lowest Density [mg cm^{-3}]
Aerogel	[8]	1.4
Aerogel	[11]	0.6
Cellular-Network	[12]	30.4
Foam	[10]	-
Foam	[9]	1.0
Foam	[13]	1.6
This Work	-	**0.17**

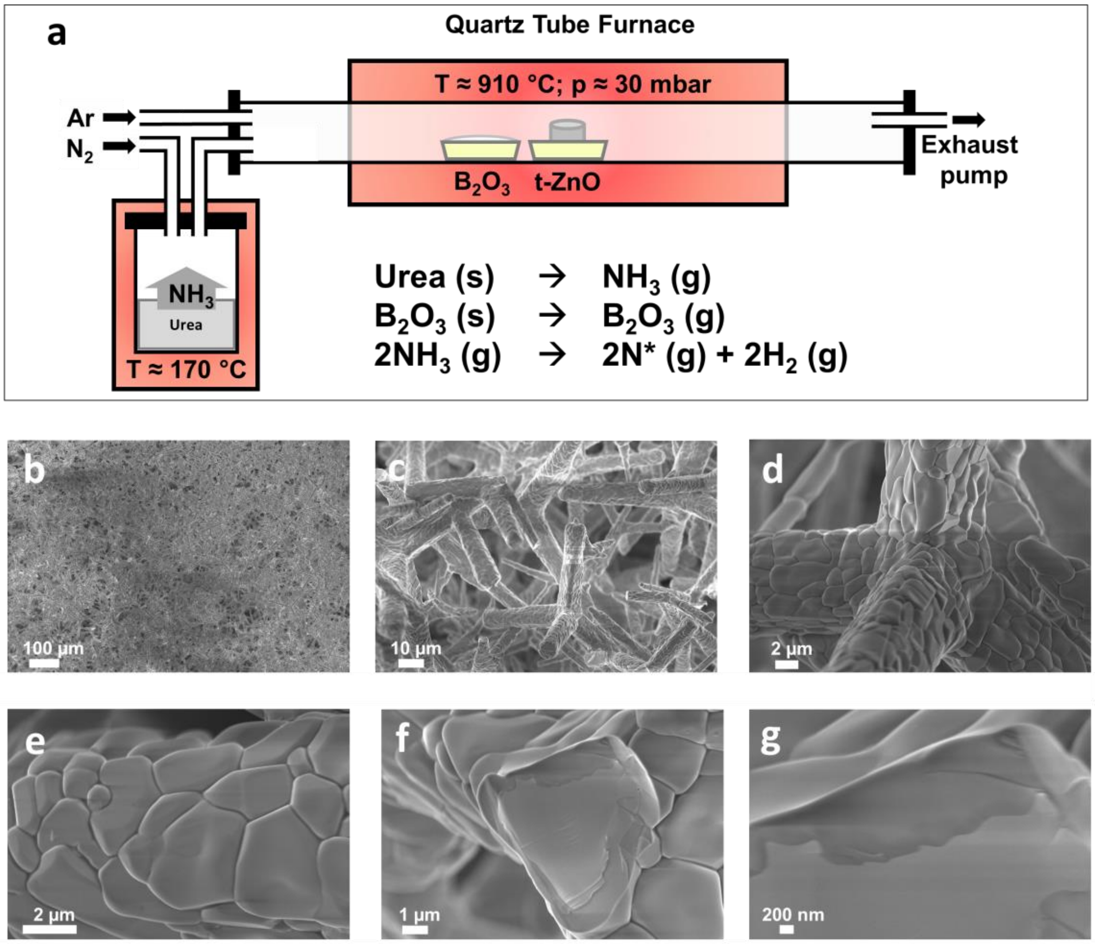

Supplementary Figure 3. a) Schematic CVD setup used for the synthesis of Aero-BN. b)-e) SEM images of the resulting structures when no NH_3 is used, showing that the surface morphology changes compared to the pure ZnO template, by the formation of a zinc borate phase one the template surface f), g) High resolution SEM of broken microrods indicate that only a thin (< 1 µm) layer is formed around the template, consisting of several interconnected islands.

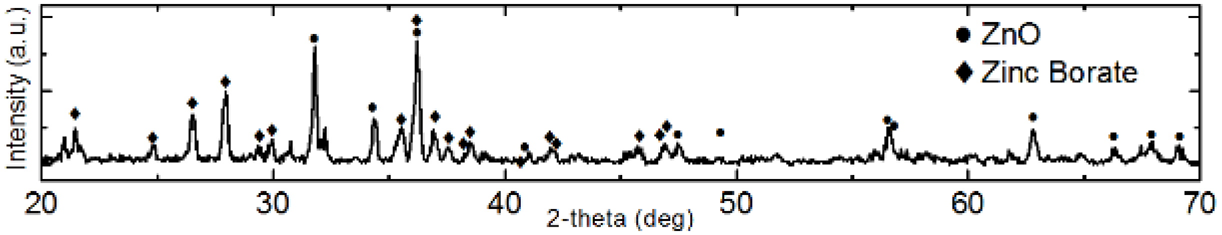

Supplementary Figure 4. XRD pattern of the reaction product formed by the reaction of ZnO and B_2O_3 indicating the formation of a zinc borate phase on top of the ZnO template.

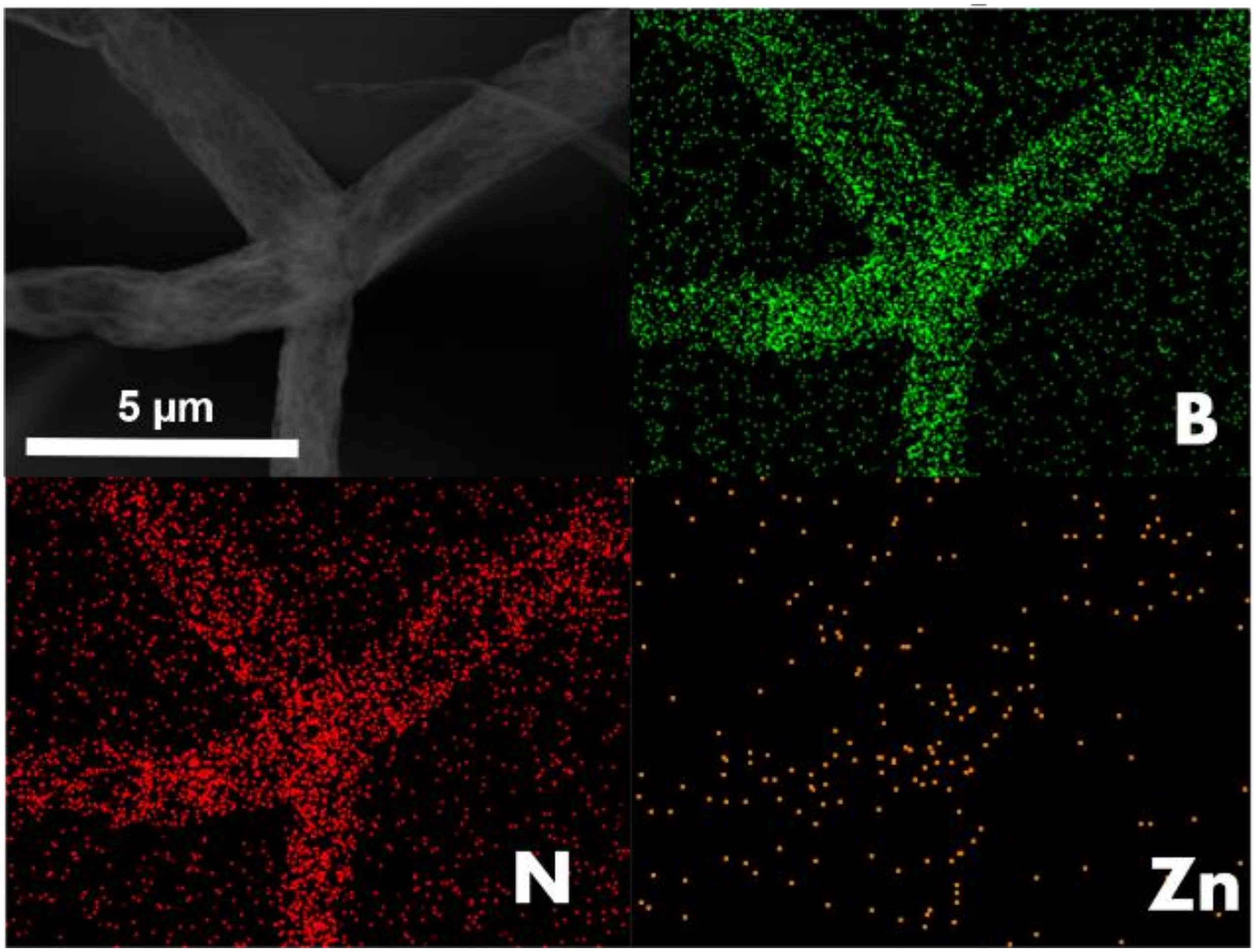

Supplementary Figure 5. Energy dispersive X-ray (EDX) measurements of Aero-BN showing that the hollow tubes consist of B and N, while no significant contribution of residual Zn (from the template) can be detected.

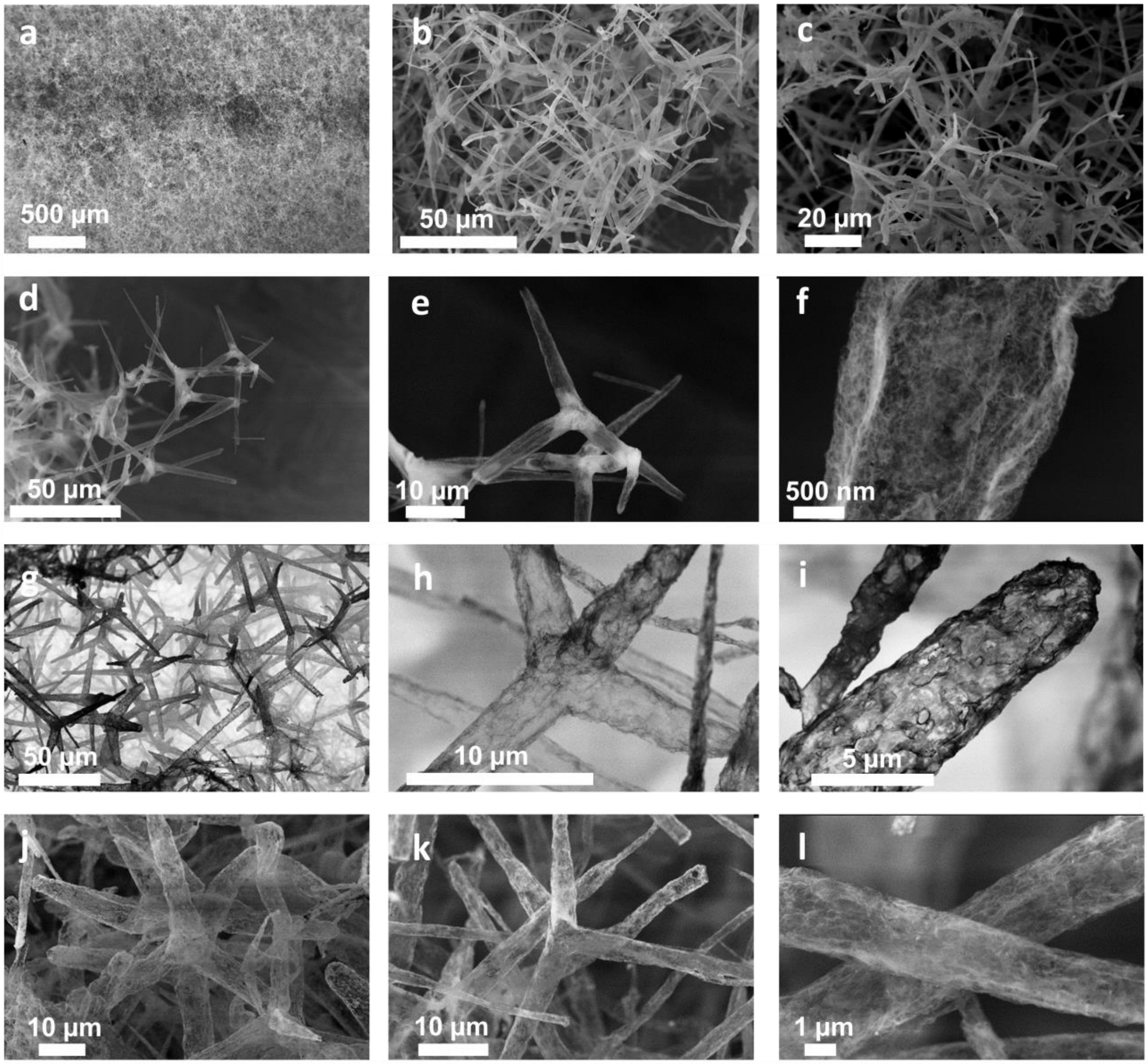

Supplementary Figure 6. a-l) SEM images of Aero-BN showing that the tetrapodal network is intact after CVD. A network consisting of interconnected hollow microtubes forms, while the template is completely removed by hydrogen. The surface of the microtubes consists of interconnected hBN platelets.

Supplementary Note 3. Evaluation of the specific surface area of Aero-BN foams and ZnO networks

The specific surface area of lightweight framework structures having a high free volume (> 90%) is difficult to obtain by standard techniques, such as BET, due to the fact that the overall amount of material per volume, thus surface area per volume, is rather low. However, since Aero-BN structures are based on a template-assisted CVD process, the specific surface area can be estimated by the following calculation. Similar calculations have already been employed for graphene foams grown by nickel-foam assisted CVD.[22]

1. Consider a ZnO template density ~ 0.3 g cm^{-3} (porosity ~ 94 %).
2. Assuming 1 cm^3 volume, we get a mass of ZnO ~ 0.3 g.
3. Dividing by the density of ZnO ~ 5.61 g cm^{-3} we obtain a volume filled with ZnO ~ 0.053 cm^3.
4. The template consists of rods (filled) with a mean diameter ~ 3 µm and a length ~ 27 µm. Thus each rod has a volume ~ $1.908 * 10^{-10}$ cm^3.
5. Dividing the volume of ZnO in the template (0.053 cm^3) by the volume of a rod we get the total number of rods ~ $2.8 * 10^8$.
6. Each rod has a surface area (assuming a smooth surface) ~ $2.615 * 10^{-10}$ m^2, resulting in a total surface area ~ 0.073 m^2 per cm^3 of template, giving a specific surface area ~ 0.244 m^2 g^{-1}.
7. Since the Aero-BN is based on these templates, consisting of hollow tubes with nanoscopic wall-thickness instead of filled rods, we can assume that the number of tubes is equal to the number of rods. As a rough estimation, the surface area can be taken double, due to the fact that we have hollow tubes instead of rods.
8. The total volumetric surface area is thus ~ 0.1465 m^2 cm^{-3}. Normalizing this by the density of the macroscopic Aero-BN network (e.g. 0.17 mg cm^{-3}) we get a specific surface area ~ 862 m^2 g^{-1} in the case of Aero-BN. However, due to the fact that the SEM images indicate a rather rough surface structure of the hBN tubes, the above estimated surface area can be assumed as a lower limit.

Supplementary Note 4. Additional TEM investigation of hollow hBN microtubes

The σ^* and π^* features seen in the EEL spectra (**Supplementary Figure 7**) can be assigned to specific orbitals and their relative geometric orientation has been intensively studied by angle and orientation dependent EELS, XAS and XPS.[23–27] We acquired EELS spectra on the edges, central and intermittent positions of an Aero-BN arm (**Supplementary Figure 7**). The intensity of π^* features is lowest when the BN (0001) plane is perpendicular to the beam (i.e. the p_z orbital is parallel to the beam; position 3) and highest when the c-plane is parallel to the beam, consistent with Ref.[23]. While the excitation of a B-1s core-electron is easiest when the momentum of the incoming electrons is parallel to the long axis of the orbital, this is only observable if both convergence and collection semi angle are small. At high collection angles the relative intensity of σ^* and π^* reverses, as seen in **Supplementary Figure 7**.

High resolution micrographs highlight the existence of numerous point and triangle defects as shown in **Supplementary Figure 8**. Ref.[28] suggested that these defects can have diverse influence on magnetic and electronic properties. Applying an average background subtraction filter (ABSF) to high resolution micrographs can improve the image quality by removing noise[29], enhancing crystalline regions through their FFT, **Supplementary Figure 8a**. This also minimizes the visibility of defects, such as the point and triangle defects in **Supplementary Figure 8**. Removing the ABSF processed image from its original micrograph makes these defects stand out and removes all crystalline areas. As result, the path of the surface step between BN of different thicknesses marked by yellow arrows in **Supplementary Figure 8** is more apparent. The same holds for the contaminations or amorphous areas in the top right as well as all the point and triangle defects. The FFT in **Supplementary Figure 8b** shows a BN domain rotated by ~26.5° with respect to the predominant orientation. This rotation by an angle other than 60° seems to occur on top (or

below) of the predominant domain with the two domains exhibiting parallel c-planes and the BN sheets of different orientation lie on top of each other.

The wall thickness of Aero-BN is determined via the EELS log-ratio method[30], i.e. by comparing intensities of the elastic peak with that of the first plasmon peak (see **Supplementary Figure 9**). This yields ~ 8 nm total cross-section thickness. As Aero-BN is a hollow structure, the thickness per wall is half of that, giving a wall thickness ~ 3-4 nm. In general, the thickness determined by the EELS log-ratio method varies between different areas and lies between 4 and 25 nm. While the calculated thickness ~ 4 nm per wall seems to be thin, comparison of bright field contrast between lacey carbon support and Aero-BN suggests that Aero-BN is even thinner than lacey carbon. This is between 10 and 30 nm thick[31]. Thus, the calculated ~ 4 nm thickness in some areas of Aero-BN is plausible.

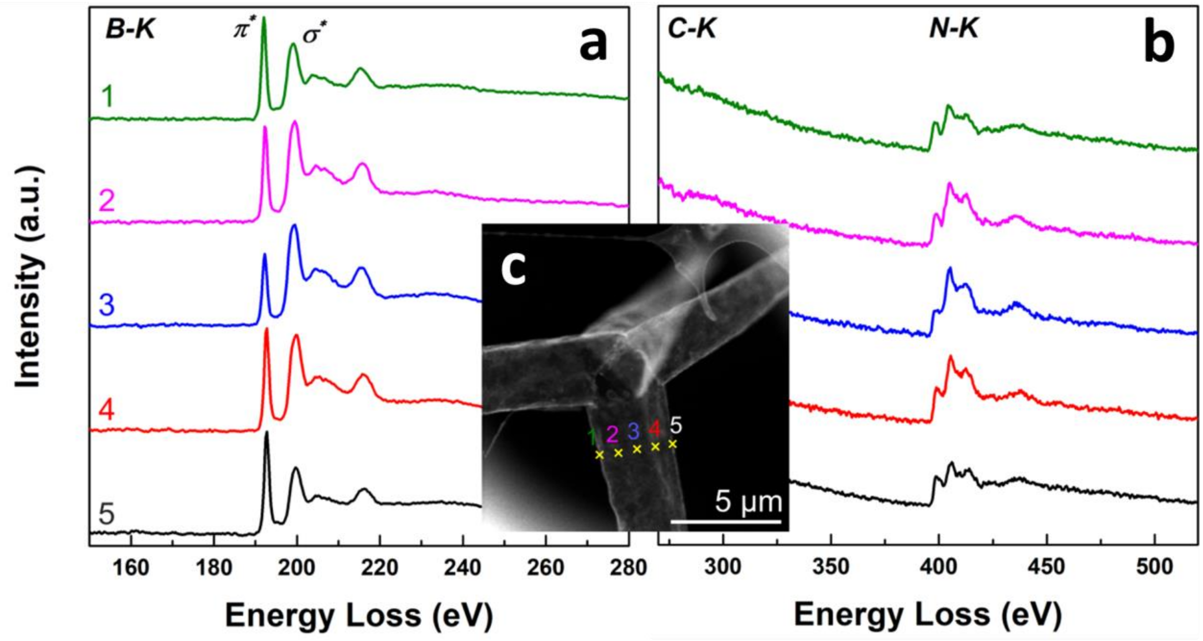

Supplementary Figure 7. Core-loss spectra of a) B and b) N at different positions c) on an Aero-BN hollow microtube. The spectra exhibit the characteristic intensity shift from $\boldsymbol{\pi}^*$ to $\boldsymbol{\sigma}^*$ when the incidence of the beam changes from parallel to the c-plane (1, 5) to perpendicular (3).

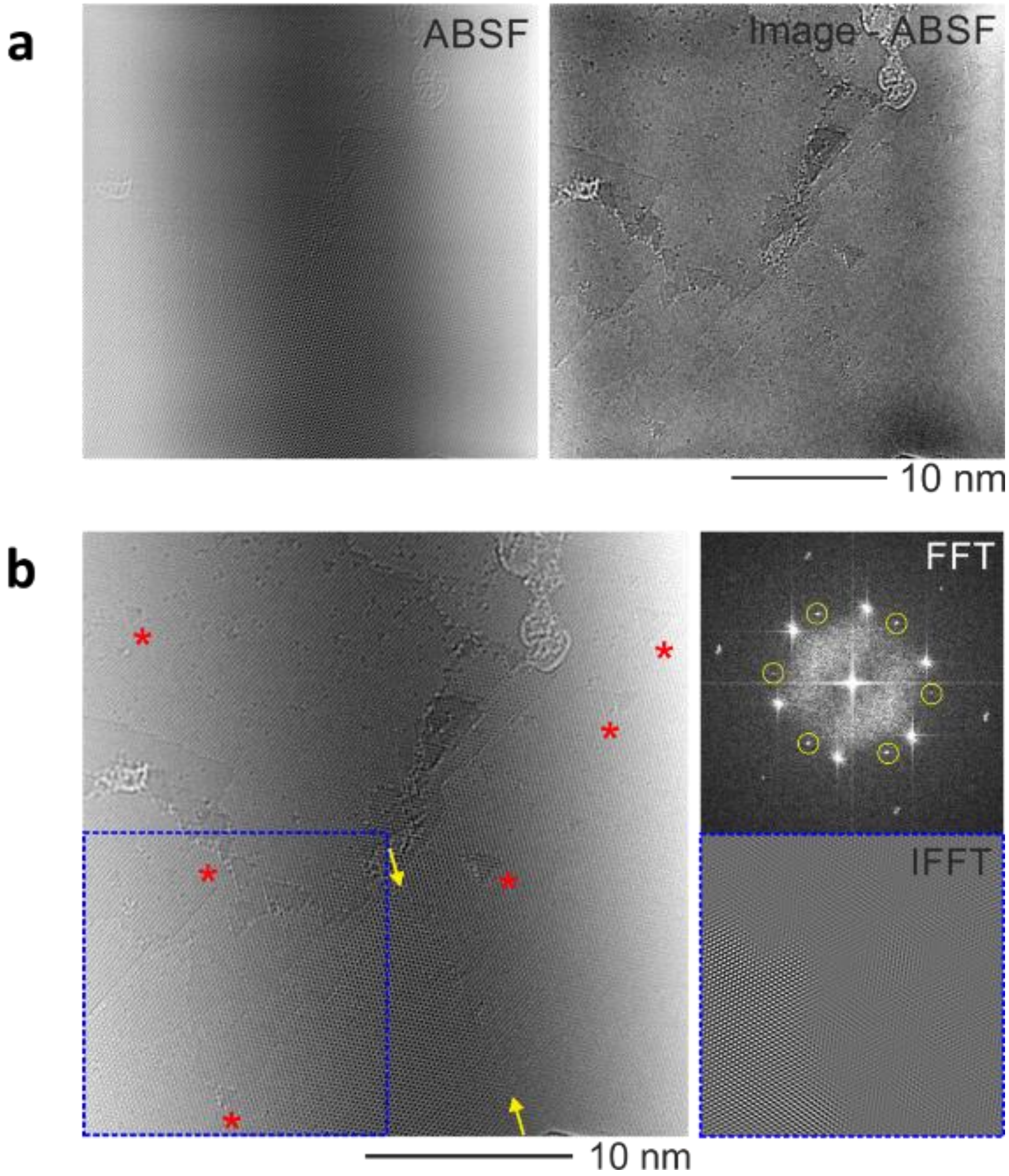

Supplementary Figure 8. a) ABSF processed image (left) and image subtracted from the original (right). The latter highlights various defects. b) High resolution micrograph of unrolled Aero-BN showcasing various defects. Several triangle defects (red asterisks) and point defects (top left region) are apparent. The yellow arrows mark an atomic ledge between sheets of different thicknesses. In the top right some amorphous areas or contaminants are visible. FFT shows that there is more than just one BN orientation. The reflections marked by yellow circles in the FFT belong to a BN domain rotated ~ 26.5 ° around the c-axis. The IFFT from these reflections shows that this domain is located in the bottom left corner (dashed blue square).

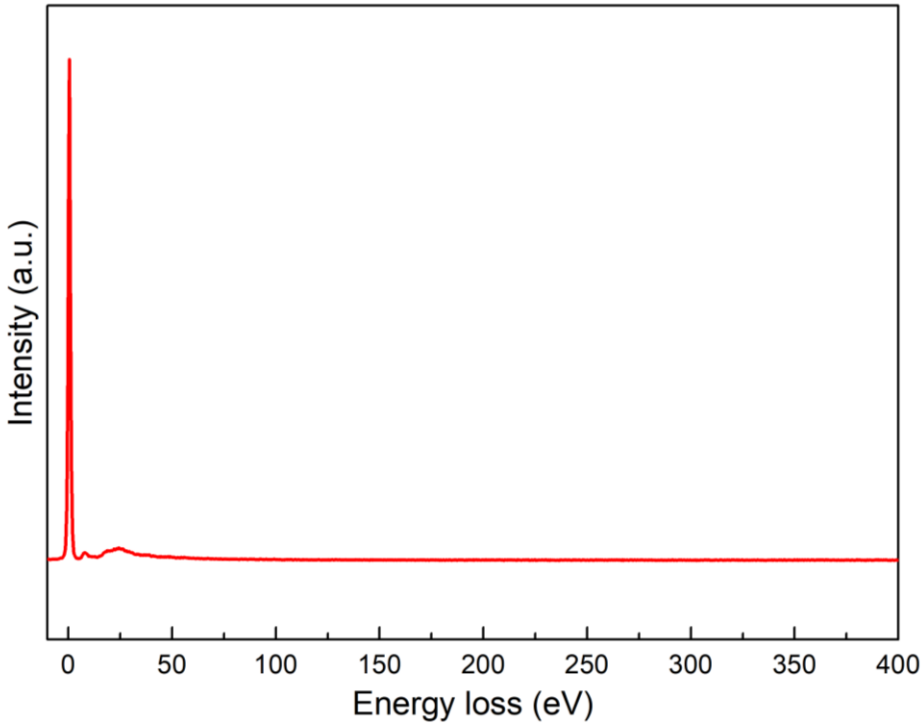

Supplementary Figure 9. EEL spectrum with ZLP and plasmon losses. The thickness calculated from this spectrum by the log-ratio method is ~ 25 nm per wall. The mean free path in BN is ~ 128 nm according to the Malis model[30].

Supplementary Note 5. Structural and material requirements for the perfect laser light diffuser

The most important requirement for an ideal optical diffuser is a low, almost negligible, absorption in the visible range, allowing for efficient light scattering. Additionally, to resist high input powers, a high laser damage threshold is required. The first two of those criteria are fulfilled in the case of an insulating ceramic material with a large band gap (at least > 3.5 eV), and can be fulfilled by a broad range of materials, including silica, hBN and others.

The light scattering properties of a diffuser are mainly determined by its micro- as well as nanostructural arrangement. Efficient light-scattering events can only be achieved by abrupt changes in the dielectric constant during light propagation.[32] This can be achieved using a material which is anisotropic on the wavelength scale, e.g. due to microscopic density fluctuations.[32,33] This is a well-studied phenomenon in transparent silica based aerogels[32], in which particle agglomerates on the micrometer scale cause unwanted Rayleigh scattering events, resulting in a limited optical transparency.[32–35] Typically silica aerogels are based on a dendritic microstructure with apparent densities between 0.03 and 0.35 g cm^{-3}, a mean pore diameter ~ 20 nm and a primary particle diameter ~ 2-5 nm.[36] **Supplementary Figure 16a** illustrates the interaction between a typical silica aerogel with a laser beam. Due to the fact that all features of the structure are smaller than the wavelength in the visible range, the material is optically isotropic, with a mean refractive index close to 1 (air).[34,36] Therefore, the beam is almost directly transmitted through the material with only small light-matter interactions and, thus, loss in intensity.

Consequently, to create a material that scatters the light effectively, a 3D architecture based on a ceramic material has to be designed with randomly distributed features (disorder[37]) as well as feature-to-feature sizes larger than the wavelength used. These requirements can be fulfilled by several structures, e.g. by a highly porous (~ 94%) network formed of randomly distributed and interconnected zinc oxide (ZnO) microrods with several micrometers (~25 µm) in length and ~ 300 to 3000 nm in diameter. These networks are also used as the templates for the Aero-BN. Thus, both of them have the same microstructure. The arrangement of interconnected ZnO microrods results in optical anisotropy, leading to abrupt changes in the refractive index (from 1 to 2.1) during illumination, thus a high amount of light-matter interaction (e.g. scattering).

Supplementary Figure 16b shows the schematic interaction of such a network during illumination for a single light beam. Under the assumption, that almost no light is absorbed by the microrods, there are two possible mechanisms to transport the light deep into the structure: ballistic (mainly diffuse) reflection or transmission.[39] Both depend mainly on the individual thickness and optical properties of the microrod, thewavelength and incident angle. If a light ray impinges on a surface of a rod, it splits into a reflected and a transmitted beam, creating so-called primary scattered beams.[39] Those primary scattered beams will collide again with another rod, generating even more secondary scattered beams, which will follow the same mechanism, thereby splitting the beam more and more , thus diffusing the incoming light (see **Supplementary Figure 16b**). Due to the random arrangement of the rods, the ratio between reflected and transmitted light will vary strongly at each scattering event (according to Fresnel's law). However, on average the intensity of the transmitted light, I_T, will be larger than that of the reflected light, I_R. For rods having a diameter between 200 - 1000 nm simulations reveal that the mean reflection (average over all incident angles) is between 20% and 45% for ZnO rods in the visible range (see **Supplementary Figure 13**). The high amount of reflection results in a strong decrease in the light intensity over the sample length (see **Supplementary Figure 14**). Thus, even though the requirement of optical anisotropy is fulfilled and the free volume is as high as 94%, a macroscopically expanded 3D network consisting of such a microrod configuration will mainly scatter the light back in the direction of the incoming laser beam (see also **Supplementary Figure 15**).

One possibility to maintain the optical anisotropy on the wavelength scale. but changing the light scattering behavior, is to exchange the microrods by hollow ceramic microtubes with walls of deep-subwavelength dimensions (few nm), as illustrated in **Supplementary Figure 13a**. As a result of the nanoscopic walls the light scattering behavior will be dominated by Rayleigh lightscattering rather than Mie scattering, resulting in scatter events with no preferential scatter direction.[39] Furthermore, the nanoscopic wall-thickness promotes a mean reflection well below 15% in the visible range (see **Supplementary Figure 13a**). Thereby, at each light scattering event I_T will be much larger than I_R, ensuring that the light can penetrate deep into the aeromaterial structure before being completely scattered, as shown in **Supplementary Figure 16c**. This results in more effective beam splitting, thus in a higher amount of scattering events in the 3D structure

before the light leaves the structure again, producing a nearly constant light emission in all directions, as is shown in the main manuscript (Figure 4, see also **Supplementary Figure 15**).

We use hBN to demonstrate this, however also other lightweight materials that fulfill the above mentioned requirements can be used.

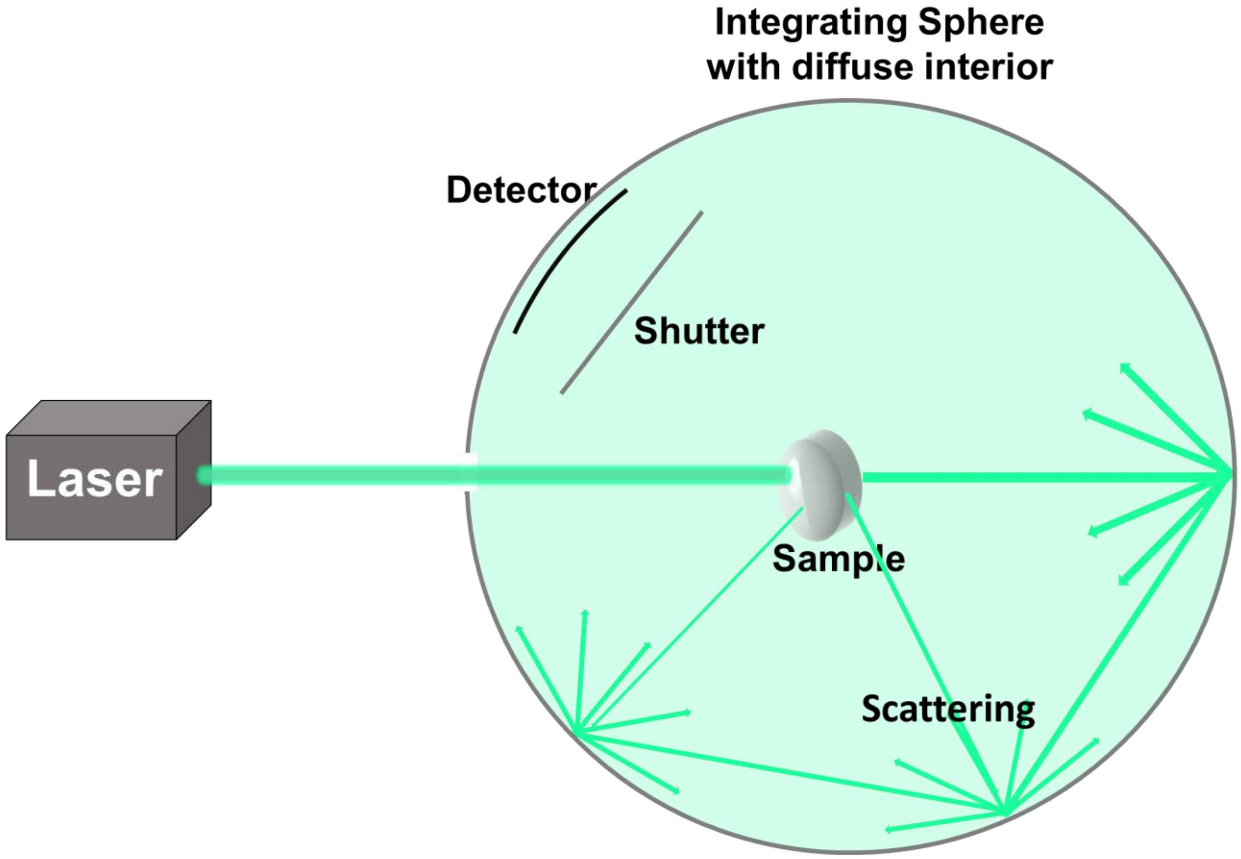

Supplementary Figure 10. Schematic setup of the absorption measurements using an integrating sphere. The sample is placed in the middle of the sphere using a thin (1 mm diameter) and polished Al rod. The laser illuminates the sample through a tiny (4 mm diameter) hole in the sphere and the flux is measured using a photodetector.

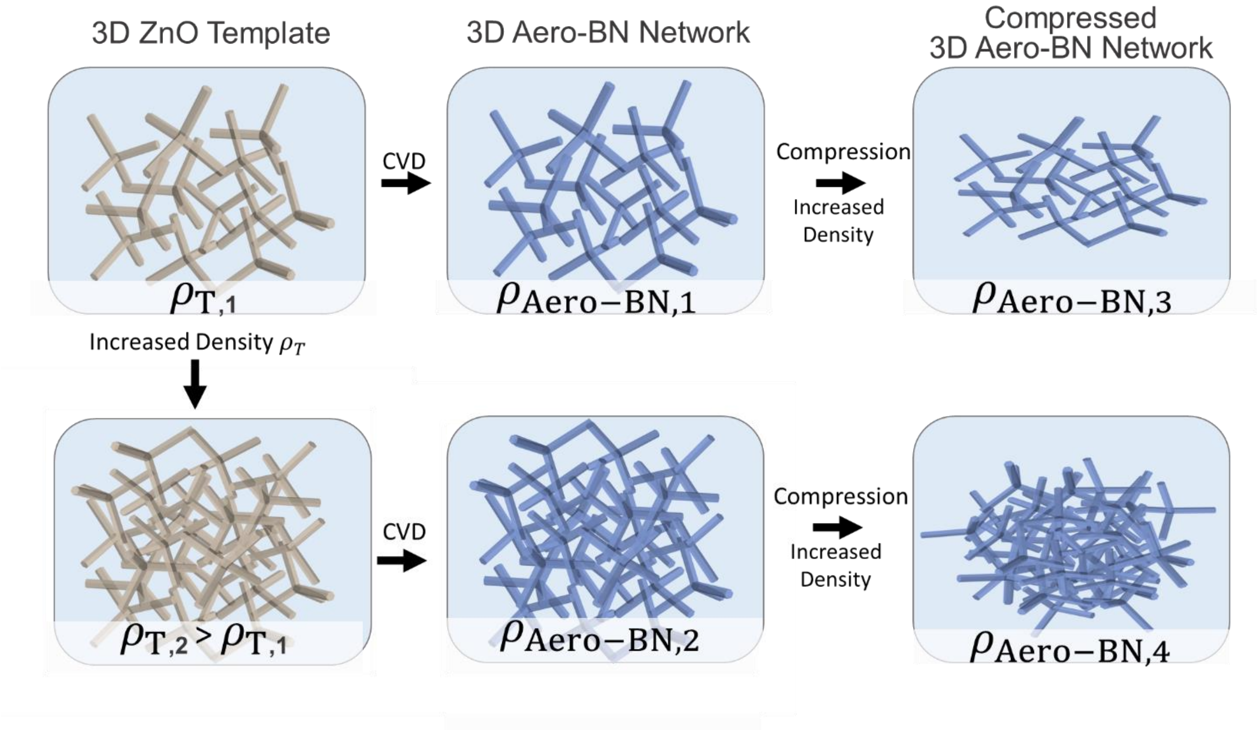

Supplementary Figure 11. Control of Aero-BN density: By changing the initial template density ($\boldsymbol{\rho}_{T}$) by controlling the mass of ZnO as well as the volume of the mold during template fabrication, the density of the Aero-BN network ($\boldsymbol{\rho}_{\text{Aero-BN}}$) can be tailored, resulting in a nearly isotropic network of interconnected hollow microtubes. By further compression (or even elongation) $\boldsymbol{\rho}_{\text{Aero-BN}}$ can be further tuned. In the case of a monoaxial compression, this results in an anisotropic change in the network volumetric and mechanical properties.

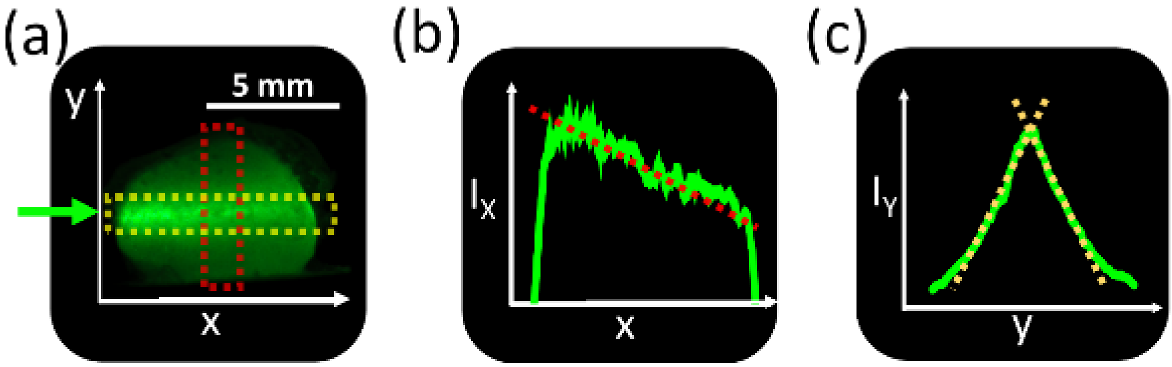

Supplementary Figure 12. a) Photograph of a sample with $\boldsymbol{\rho}_{\mathbf{Aero-BN}}$ ~ 0.17 mg cm^{-3} illuminated with a 100 mW laser (spot size ~1 mm) at 520nm. b,c) Intensity plots in *x* and *y*-direction of the photograph shown in a). The dotted lines illustrate the almost linear decrease in intensity along *x* and *y* directions.

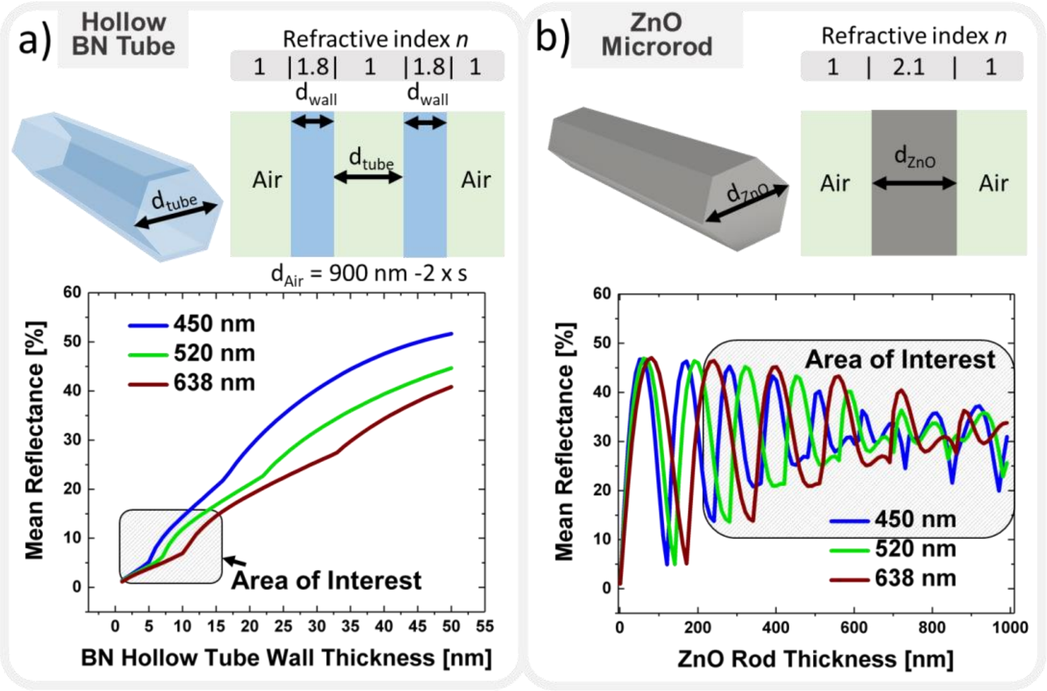

Supplementary Figure 13. Theoretic reflectance calculations. a) Mean reflectance vs. hollow tube wall thickness (d_{wall}) for a hollow hBN tube with a total diameter of 920 nm for different wavelengths. The diameter of the tube is d_{tube}. b) Mean reflectance vs. ZnO rod thickness (d_{ZnO}) for different wavelengths. The area of interest marks the regions relevant for our work. The calculations are based on those of Ref.[38]

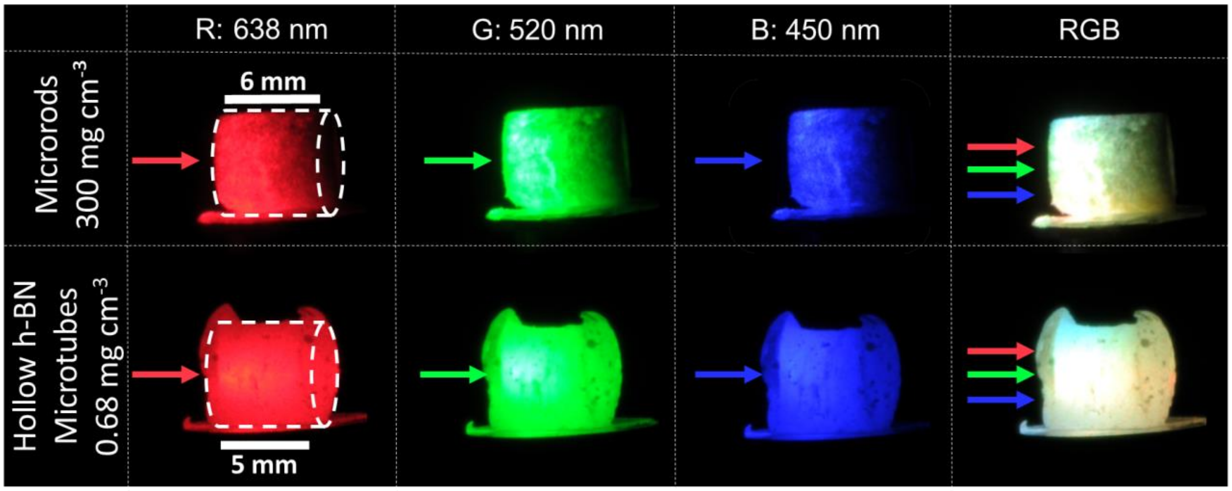

Supplementary Figure 14. Photographs of a cylindrical ZnO microrod network (density ~ 0.3 g cm^{-3}; porosity ~ 94 %) and a hollow hBN microtube network (density ~ 0.68 mg cm^{-3}; porosity > 99.99 %) illuminated at ~ 100 mW at different wavelengths. When all lasers are used (RGB), the light is mixed giving white illumination. The laser beams are focused on the middle of the sample. The direction of the laser beams is indicated by arrows.

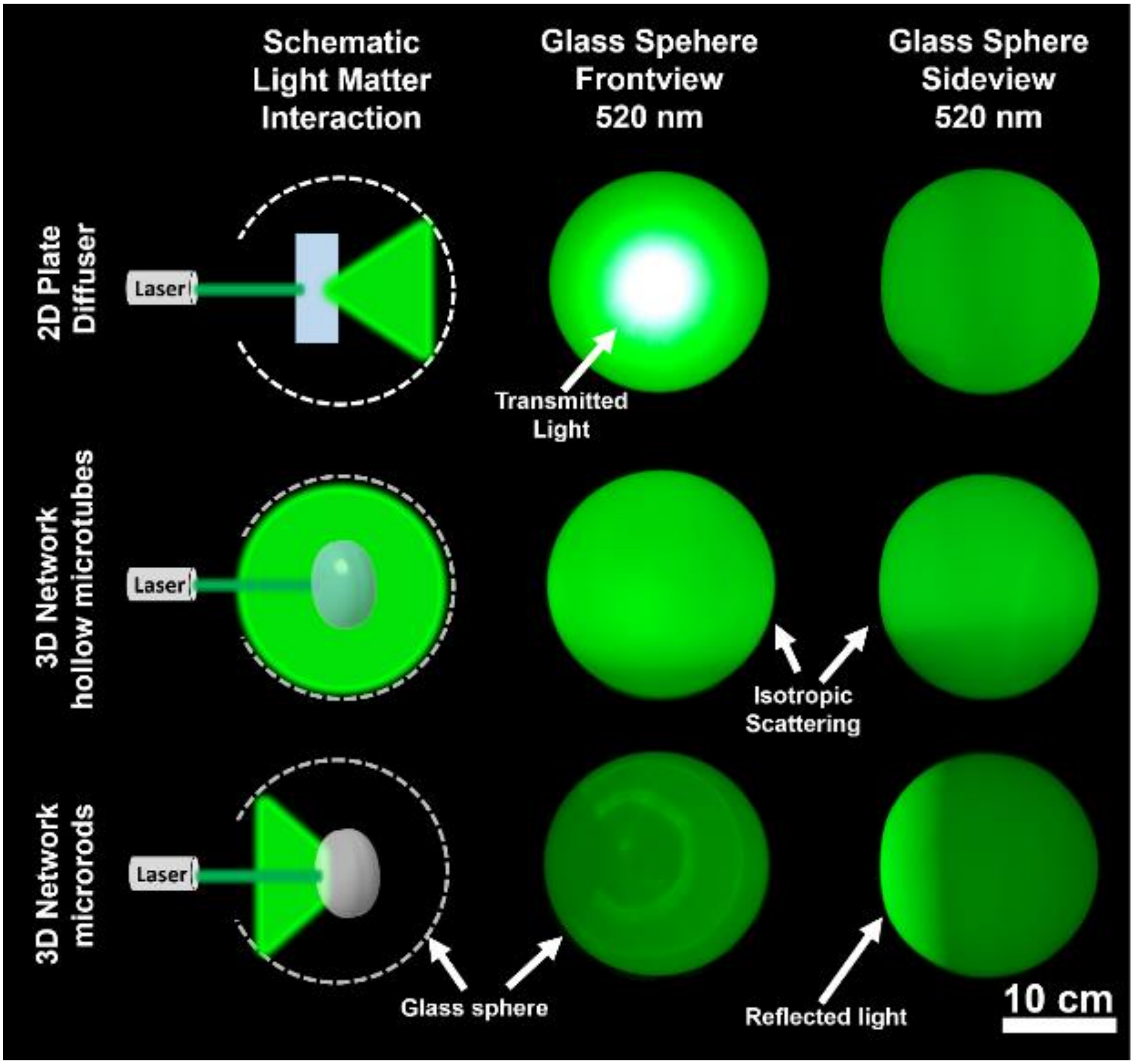

Supplementary Figure 15. Schematic of light scattering from a commercial available plate diffuser, a 3D network of interconnected hollow hBN microtubes (density ~ 0.68 mg cm^{-3}, porosity > 99.99%) and a 3D network of interconnected ZnO microrods (density ~ 0.3 g cm^{-3}, porosity ~ 94%). The photographs show the light distribution when illuminated under a semitransparent glass sphere (diameter ~ 20 cm; front and side view) at 100 mW for 520 nm. Only the 3D network of hollow hBN microtubes results in a homogeneous light distribution represented by a homogeneous glow of the glass sphere, whereas in the case of the plate diffusor most light is transmitted or scattered only in a small (< 30°) angular range. The 3D ZnO microrod network shows a visible high degree of reflectance, with no light transmitted.

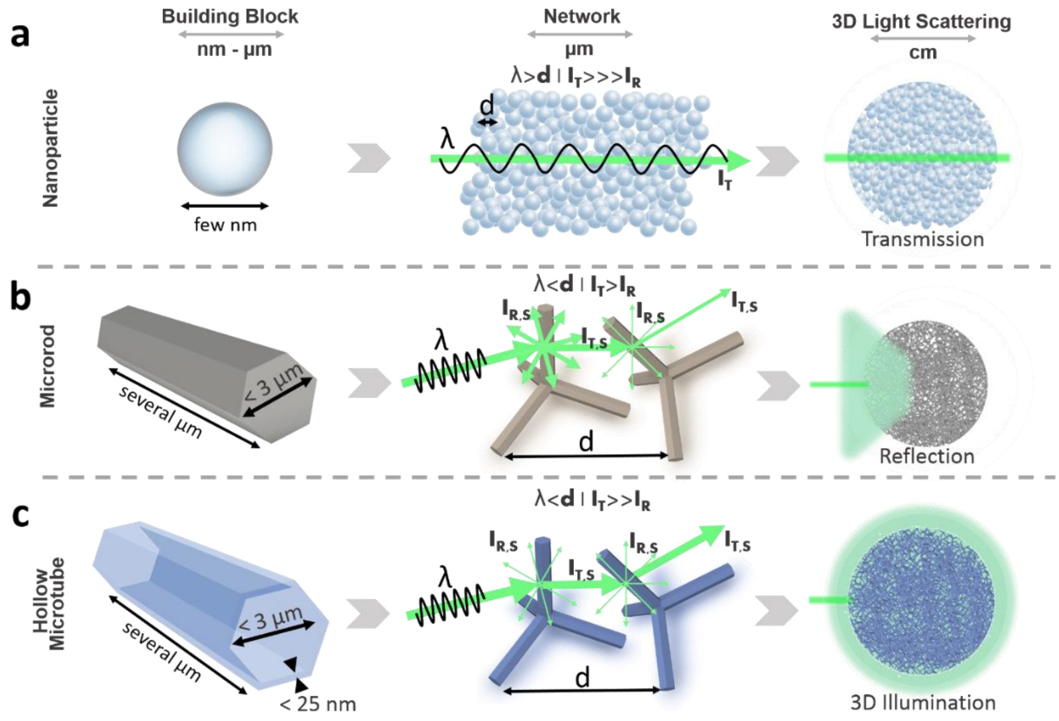

Supplementary Figure 16. Schematics of 3D ceramic nanoarchiectures based on different individual building blocks and their interaction with light: a) Optically isotropic material (silica aerogel) based on spherical nanoparticles: nearly no light-matter interaction due to features sizes smaller than the wavelength of visible light, the primary light beam is completely transmitted through the material. b) Optically anisotropic material consisting of interconnected microrods. The mean distance d between the microrods is several times the wavelength λ, resulting in sharp changes in the refractive index, thus a high amount of light scattering events. The primary light beam is split into a reflected/scattered (R,S) and transmitted beam (T) with the intensity of the transmitted beam being larger compared to that of the scattered/reflected beam $I_{T,S} > I_{R,S}$ at each scattering event. As a result, the 3D macroscopic expanded structure will mainly scatter the light back in the direction of the incoming laser beam. c) Optically anisotropic material consisting of hollow interconnected microtubes (wall thickness between 3 - 25 nm) having the same dimensions as the microrod network. The nanoscopic walls allow for Rayleigh scattering events resulting in a much lower reduction in light intensity over the sample dimensions and thus in a homogeneous 3D light distribution of the macroscopic expanded 3D network.

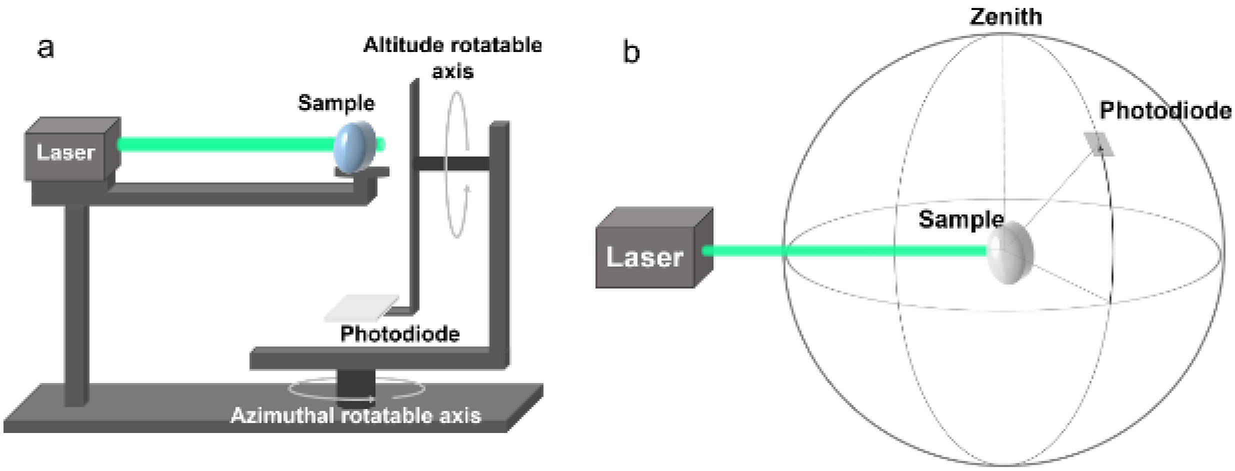

Supplementary Figure 17. a) Schematic photo-goniometer used to measure the light scattering. The photodiode is mounted so that it can be rotated around the sample on the surface of a sphere, as shown in b), thereby the photocurrent produced by the scattered light can be measured in all directions.

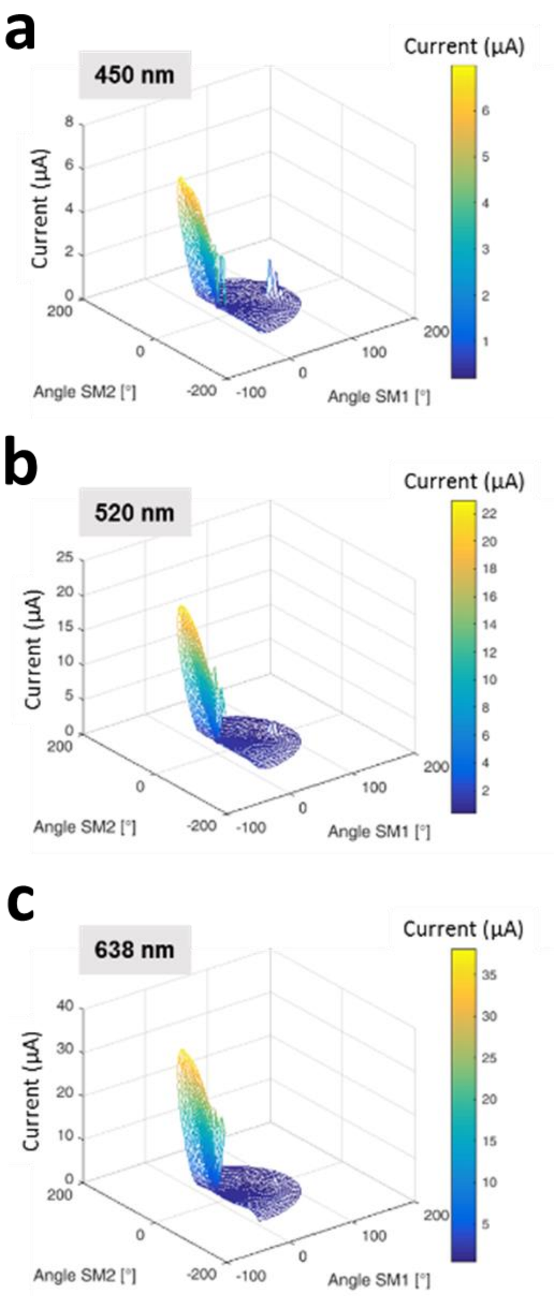

Supplementary Figure 18. Polar plots of angular dependent photocurrent for a 3D ZnO network consisting of interconnected microrods ($\boldsymbol{\rho}_{\mathbf{t-ZnO}}$ ~ 300 mg cm^{-3}) measured under laser illumination at a) 450, b) 520 and c) 638 nm, respectively, with a photogoniometer, as shown in **Supplementary Figure 17**.

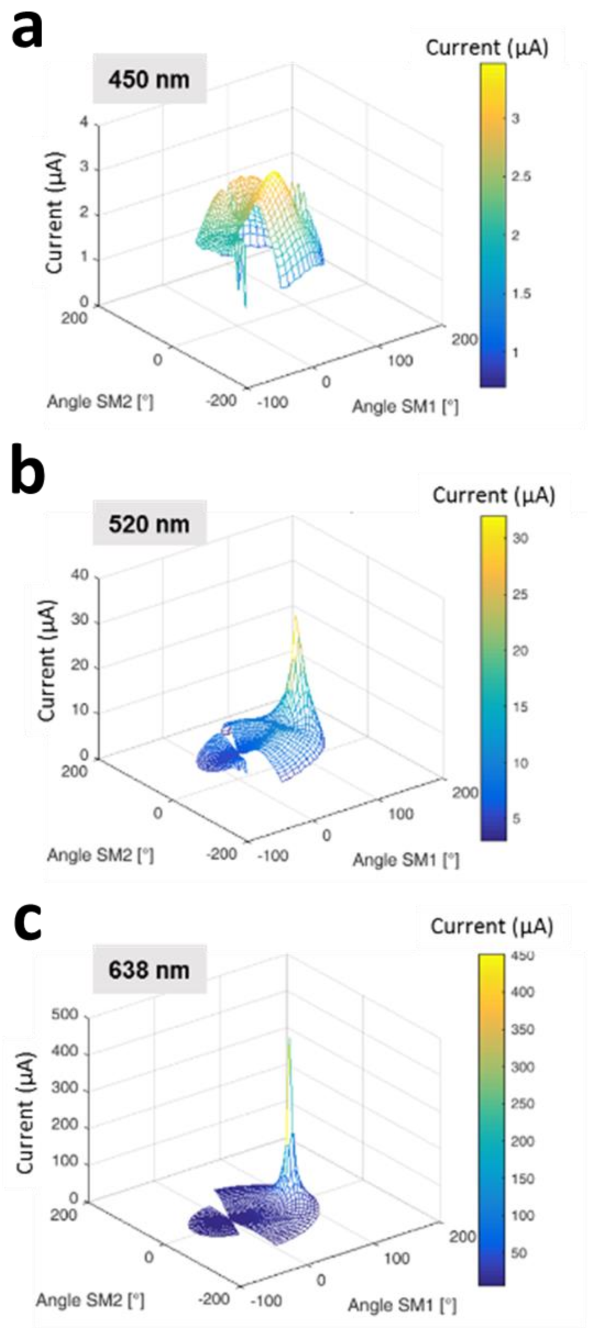

Supplementary Figure 19. Polar plots of angular dependent photocurrent for a 3D Aero-BN network consisting of interconnected hollow microtubes ($\boldsymbol{\rho}_{\mathbf{Aero-BN}}$ ~ 0.17 mg cm^{-3}) measured under laser illumination at a) 450, b) 520 and c) 638 nm, respectively, with a photogoniometer, as shown in **Supplementary Figure 17**.

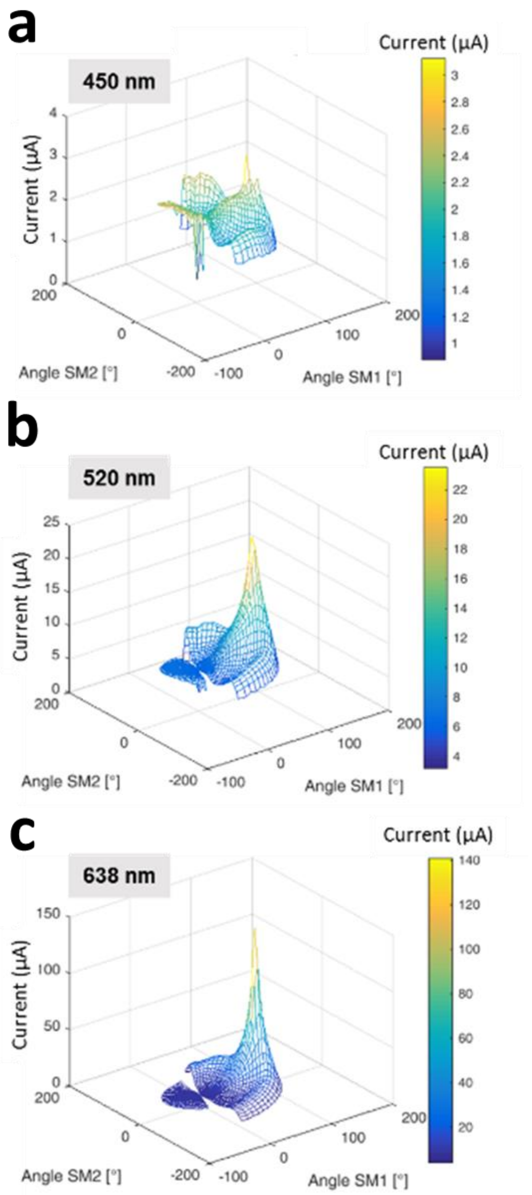

Supplementary Figure 20. Polar plots of angular dependent photocurrent for a 3D Aero-BN network consisting of interconnected hollow microtubes ($\boldsymbol{\rho}_{\mathbf{Aero-BN}}$ ~ 0.68 mg cm^{-3}) measured under laser illumination at a) 450, b) 520 and c) 638 nm, respectively, with a photogoniometer, as shown in **Supplementary Figure 17**.

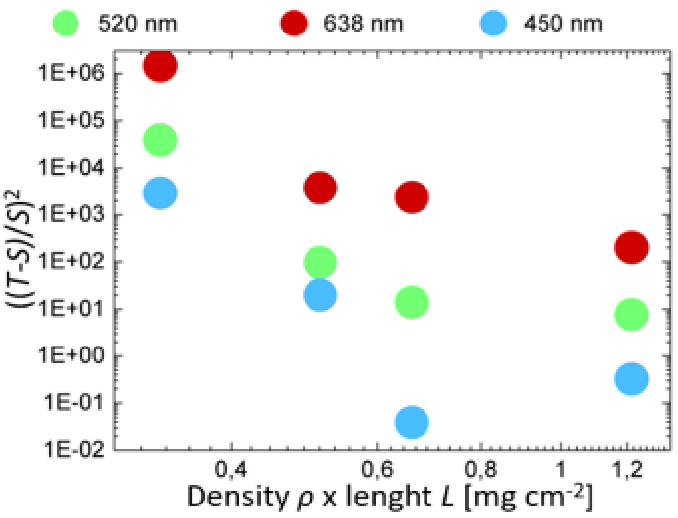

Supplementary Figure 21. Relative deviation from the average scattering (*S*) with respect to the transmitted light (*T*) versus the optical areal density (density times thickness) for three wavelengths. The error-bar is within the size of the symbols.

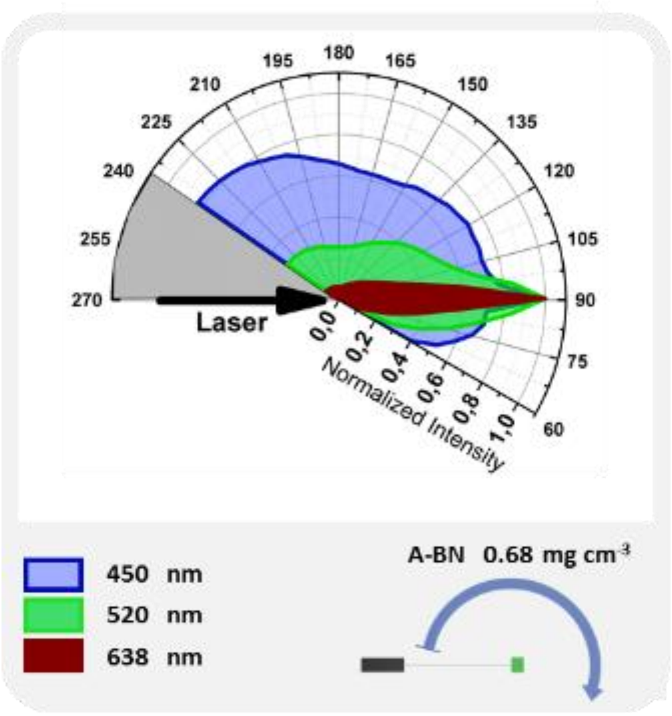

Supplementary Figure 22. Normalized photocurrent dependence with respect to an azimuthal rotation of the photodiode for different wavelengths. The density of Aero-BN is ~ 0.68 mg cm^{-3}. Each laser has a power of ~ 100 mW.

Supplementary Note 6. Light scattering mechanism in Aero-BN

To quantify the light scattering mechanism in dependence of the Aero-BN density, we studied the transmission as a function of monoaxial compression, resulting in a $\rho_{\mathrm{Aero-BN}}$ increase as shown in **Supplementary Figure 23**. The specimen has a length L = 6 mm and is compressed orthogonally to the laser beam. The total ballistic transmission T is measured using a continuous laser at 520 nm and 100 mW and an integrating sphere. The initial $\rho_{\mathrm{Aero-BN}}$ ~ 0.37 mg cm^{-3} is increased by compression to ~ 0.93 mg cm^{-3}. The equivalent Poisson's ratio ν (transversal relative expansion divided by the amount of monoaxial compression strain) of Aero-BN is, even up to large compressive strain, estimated to be close to zero, as measured from compression experiment simulations on our specific network (see FEM simulation results and **Supplementary Figure 24**). This is a common characteristic for aero-materials.[40]

Therefore, the sample lengths L_x and L_y, orthogonal to the compression in z, remain almost constant during compression, and can thus be excluded as cause for any transmission changes (see scaling for monoaxial compression in **Supplementary Table 3 and Supplementary Figure 24**). The compression irreversibly deforms the network structure, however the microtubes do not break due to their nanometer sized wall thickness and consequent flexibility, as was previously shown for aerographite networks[41] having a similar geometry and mechanical properties. As the network is compressed, $\langle d_{\mathrm{MT}} \rangle$ decreases up to 10 µm in z-direction (see computation by FEM simulation in **Supplementary Figure 25**), thereby increasing the volumetric and optical density. According to the Ioffe-Regel criterion[42], which describes the ratio of the photon wave-vector k to the mean free path l^*, in such domains (where $kl^*<1$) the light transport properties can change from normal diffusive (random walk) behaviour to localization-based transport, meaning that photons can become trapped.[42,43]

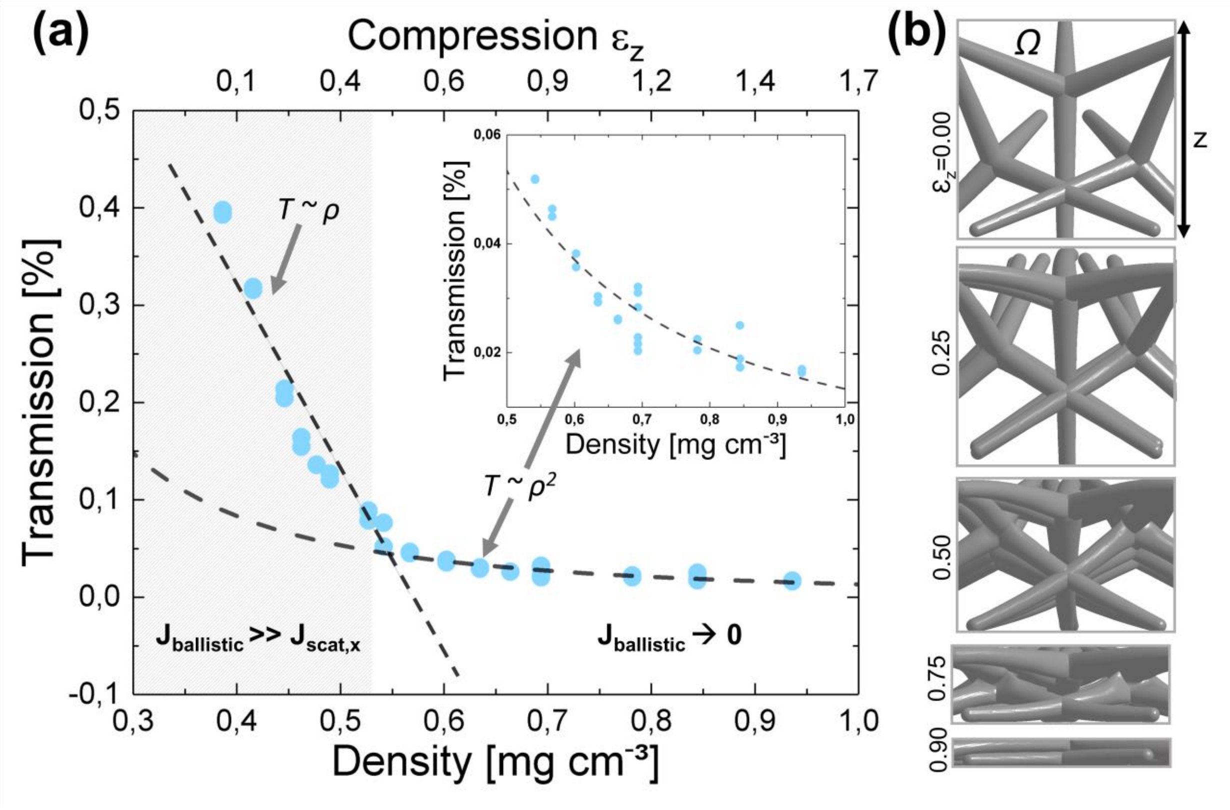

Supplementary Figure 23. Tunable Disorder: a) Transmission as a function of $\rho_{\text{Aero-BN}}$. The network density is increased by compressing the sample in the *z*-direction with compressive strain ε_z. The initial $\rho_{\text{Aero-BN}}$ ~ 0.37 mg cm^{-3} is increased up to ~ 0.93 mg cm^{-3}. The transmission shows a regime in which $T \sim \rho_{\text{Aero-BN}}$ and a regime in which $T \sim \rho^2_{\text{Aero-BN}}$ regime. The inset shows the quadratic fit. b) FEM snapshots ($\rho_{\text{Aero-BN}}$ ~ 0.376 mg cm^{-3}) at different ε_z, showing the mechanical deformation of the sample and a decrease in the projected porous area Ω.

Supplementary Figure 23a points to two different regimes. At $\rho_{\text{Aero-BN}} < 0.57$ mg cm^{-3}, we have a linear decrease in T as a function of $\rho_{\text{Aero-BN}}$, whereas for $\rho_{\text{Aero-BN}} > 0.57$ mg cm^{-3} we observe a quadratic reduction in the T. No exponential decay of T, as a function of $\rho_{\text{Aero-BN}}$ is found, meaning that only a negligible amount of light is absorbed by the specimen, as by the Beer-Lambert law[44], which describes the attenuation of light through an absorbing medium, is never reached.

The data in **Supplementary Figure 23a** can be understood by a diffusion model for the scattered photons assuming negligible absorption losses within the sample, i.e. $-\nabla\vec{J} = 0$ (continuity equation[44] for steady state) with a current $\vec{J}$ that can be described by the following differential equation representing an inhomogeneous diffusion[44]:

$$\vec{J} = -\left(D_x \frac{\partial n}{\partial x}, D_y \frac{\partial n}{\partial y}, D_z \frac{\partial n}{\partial z}\right)\left[= \frac{\nu_\mathrm{e}}{2A} n_i = K\, n_i\right] = 0 \qquad \text{(S1)}$$

where $\vec{J}$ is the photo current density vector, D_x, D_y, D_z being the diffusion constants in *x*-, *y*-, and *z*-direction, *n* the photon density, ν_e the energy velocity at a certain wavelength, which is given by the vacuum speed of light c_vac divided by the refractive index *n* ($\nu_\mathrm{e} = c_\mathrm{vac}/n$), *A* is a dimension-less coefficient representing Fresnel effects at the interface to the surrounding and *K* being the photon escape velocity.[44] The square brackets shown in **Supplementary Equation 1** describe the boundary condition, reflecting the photon conservation at the interface between the sample and the surrounding.[44] Taking the above boundary condition and a cubic geometry, the solution of the homogeneous differential equation can be written by the separation approach:

$$n(x, y, z) = f(x) \times g(y) \times h(\mathrm{z}) \qquad \text{(S2)}$$

where the 3 functions (*f*(*x*), *g*(*y*), *h*(*z*)) are linear. This leads to a linear decrease of the photon concentration in all directions from the surface of the incident laser beam to all other surfaces. For the inhomogeneous differential equation, two cases can be considered. First, for l*~L the conversion of ballistic to scattered photons through the sample can be approximated by a constant a in the *x*-direction, leading to a quadratic function for the solution of the inhomogeneous differential equation $-\nabla\vec{J} + a = 0$. Second, if $l^* << L$, the whole laser beam will be scattered close to the incident surface, which can be approximated by a constant surface current density as a boundary condition for the homogeneous solution.

For a specimen with ν ~ 0, a monoaxial compression in *z*-direction does not change the light scattering properties in *z*-direction since *L* and *l** will both change with the same order, but leads to an increased scattering in *x*- and *y*-directions by placing more scattering centres into the path of the photons as illustrated in **Supplementary Figure 23b** by the reduced projected porous area *Ω* as a function of compression in *z*-direction (see also **Supplementary Figure 26**). This decreases the diffusion coefficients D_x and D_y. As discussed before, the linear dependence of *T* on the density, **Supplementary Figure 23a**, and in the intensity plot over the *x*-dimension, **Supplementary Figure 12**, implies negligible absorption losses.[42]

Thus, according to the steady state continuity equation all scattered photons not leaving the sample in the *x*-direction have to leave the sample along *y*- or *z*- direction. Thus, at the transition point from the linear to the quadratic regime in **Supplementary Figure 23a** all photons are scattered at least once, i.e. no ballistic photons leave the sample in the *x*-direction. The following quadratic decay reflects the properties of the solution of the inhomogeneous differential equation (**Supplementary Equation 1**). So by monoaxial tuning of the density of scattering centres in the *z*-direction, the scattering efficiency in *x*,*y* can be enhanced with the drawback of a linear intensity decrease of scattered photons along *x*. For a specimen with a much higher initial density (e.g., $\rho_{\mathrm{Aero-BN}}$ of ~ 0.68 mg cm^{-3}) and thus higher number of scattering centres, as shown in **Figure 4c** in the main text, no ballistic beam can be observed (**Figure 4d** in the main text). The sample radiates brightly with only a slight visible intensity reduction across the *x*-direction. For a homogeneous density of scattering centres, such a linear intensity decrease, is unavoidable, since it reflects the dependence on the distance of the homogeneous diffusion. These results are schematically summarized in the model shown in **Supplementary Figure 27a-d**. Compression along *z* ($\mathcal{E}_z$) of a low $\rho_{\mathrm{Aero-BN}} < 0.5$ mg cm^{-3} sample leads to an increase of the slope of the

concentration of scattered photons C_{scat} in x, **Supplementary Figure 27a**, by transferring a larger current of photons scattered into the z ($J_{\text{out,z,scat}}$), **Supplementary Figure 27b**. This reflects the tuned diffusion in dependence of the one dimensional compression, thus different diffusion constants in x-, y- vs. z- direction. In this configuration, the photocurrent leaving the sample in the x-direction ($J_{\text{out,x}}$) comprises ballistic ($J_{\text{ballistic}}$) and scattered ($J_{\text{out,x,scat}}$) components:

$$J_{\text{out,x}} = J_{\text{ballistic}} + J_{\text{out,x,scat}} \tag{S3}$$

where the linear reduction of the ballistic component is dominant. With increasing ε_z, the ballistic component of **Supplementary Equation 3** decreases until at a certain critical compression (depending on the initial $\rho_{\text{Aero-BN}}$, the tube wall thickness and diameter) nearly no photons are able to move through L without being scattered. The corresponding schematic representation of the light transport properties for the high $\rho_{\text{Aero-BN}} > 0.6$ mg cm^{-3} are shown in **Supplementary Figure 27c,d**. The higher number of scattering centres results in $J_{\text{ballistic}} \rightarrow 0$. The higher initial $\rho_{\text{Aero-BN}}$ also implies that the current of photons scattered into the z-direction ($J_{\text{out,z,scat}}$), **Supplementary Figure 27c,** is comparable to that scattered in the x-direction ($J_{\text{out,z,scat}} \sim J_{\text{out,x,scat}}$). In contrast to **Supplementary Figure 27b**, the homogeneous diffusion ($D_x = D_y = D_z$) leads to a homogenous transport in all directions, enabling an isotropic light distribution with only a small visible linear decrease in x-direction (**Supplementary Figure 27d**). By adjusting $\rho_{\text{Aero-BN}}$, e.g. by a slight z elongation of a high $\rho_{\text{Aero-BN}}$ sample, the C_{scat} slope in the x-direction can be tuned, leading to a more homogeneous scattering in the x-direction (decrease in the slope shown in **Supplementary Figure 27c**), but inducing an inhomogeneity between y- and z-directions. Equivalently, a compression in x-direction would lead to the same result, but without any difference in y and z.

Poisson's ratio, projected porous area and mean tetrapod distance

Let consider an infinitesimal volume of a linear, elastic, isotropic and homogeneous material, defined by the elastic modulus E and Poisson's ratio υ, with an initial length of the edges L_x, L_y, $L_z = L_0$ (thus its initial density is $\boldsymbol{\rho_0 = m/L_0^3}$, where m is the mass of the representative volume) and subjected to monoaxial compression in direction z. The elastic strains along the three directions are $\varepsilon_z = \Delta L/L_0$, $\varepsilon_x = -\upsilon\Delta L/L_0$, $\varepsilon_y = -\upsilon\Delta L/L_0$, with $\Delta L = L\text{-}L_0$. If the mass m is preserved during compression, we have $\Delta\rho = \Delta L^{(1\text{-}2\upsilon)}$, while the variation of the lateral area of the sample scales as $\Delta A = \Delta L^{(1\text{-}\upsilon)}$. By expressing the dimensionality of the imposed compressive stress via the parameter D -with $D = 1$ for monoaxial compression, $D = 2$ for biaxial compression and $D = 3$ for triaxial state- it is possible to generalize the previous calculations for an arbitrary compression state, yielding to the following interdependency relation between sample sizes and material density:

$$\left(\frac{\rho_0}{\rho}\right) = \left(\frac{L}{L_0}\right)^{\beta(D,\upsilon)} \quad \text{(S4.a)}$$

$$\left(\frac{A_0}{A}\right) = \left(\frac{L}{L_0}\right)^{\gamma(D,\upsilon)} \quad \text{(S4.b)}$$

$$\left(\frac{\rho_0}{\rho}\right) = \left(\frac{A}{A_0}\right)^{\delta(D,\upsilon)} \quad \text{(S4.c)}$$

where A is the area parallel to the plane of the principal stress for monoaxial and biaxial compression (orthogonal to the laser beam in our case) and either one of the faces for triaxial compression. The scaling exponents of **Supplementary Equations 4** assume the following expression as a function of the dimensionality:

$$\beta = D[1 + (D - 3)\upsilon] \quad \text{(S5.a)}$$

$$\gamma = 1 - \upsilon \ \text{(for } D = 1\text{)}, \gamma = 2 \ \text{(for } D = 2, 3\text{)} \quad \text{(S5.b)}$$

$$\delta = \frac{\beta}{\gamma} \quad \text{(S5.c)}$$

The resulting scaling laws for all cases are reported in **Supplementary Table 3**. These are in principle valid in the regime of infinitesimal strains, while a compressed network up to 50% of deformation should be considered in the finite strain regime. However, in place of the Poisson's ratio we can assume an analogous measure in the finite regime, i.e., more generally, the ratio between the lateral and the longitudinal elongations ($-e_x/e_z$, $-e_y/e_z$).[45]

Supplementary Table 3. Interdependence relations between ρ, A and L for a linear elastic isotropic material as a function of υ and D. The relations coherently yields for monoaxial and biaxial compression no variation of density (thus volume) for an incompressible material, υ = 0.5.

	$\boldsymbol{\rho, L}$	$\boldsymbol{A, L}$	$\boldsymbol{\rho, A}$
**Monoaxial (*D*=1)**	$\left(\frac{\rho_0}{\rho}\right) = \left(\frac{L}{L_0}\right)^{1-2\upsilon}$	$\left(\frac{A_0}{A}\right) = \left(\frac{L}{L_0}\right)^{1-\upsilon}$	$\left(\frac{\rho_0}{\rho}\right) = \left(\frac{A}{A_0}\right)^{\frac{1-2\upsilon}{1-\upsilon}}$
**Biaxial (*D*=2)**	$\left(\frac{\rho_0}{\rho}\right) = \left(\frac{L}{L_0}\right)^{2-4\upsilon}$	$\left(\frac{A_0}{A}\right) = \left(\frac{L}{L_0}\right)^{2}$	$\left(\frac{\rho_0}{\rho}\right) = \left(\frac{A}{A_0}\right)^{1-2\upsilon}$
**Triaxial (*D*=3)**	$\left(\frac{\rho_0}{\rho}\right) = \left(\frac{L}{L_0}\right)^{3}$	$\left(\frac{A_0}{A}\right) = \left(\frac{L}{L_0}\right)^{2}$	$\left(\frac{\rho_0}{\rho}\right) = \left(\frac{A}{A_0}\right)^{\frac{3}{2}}$

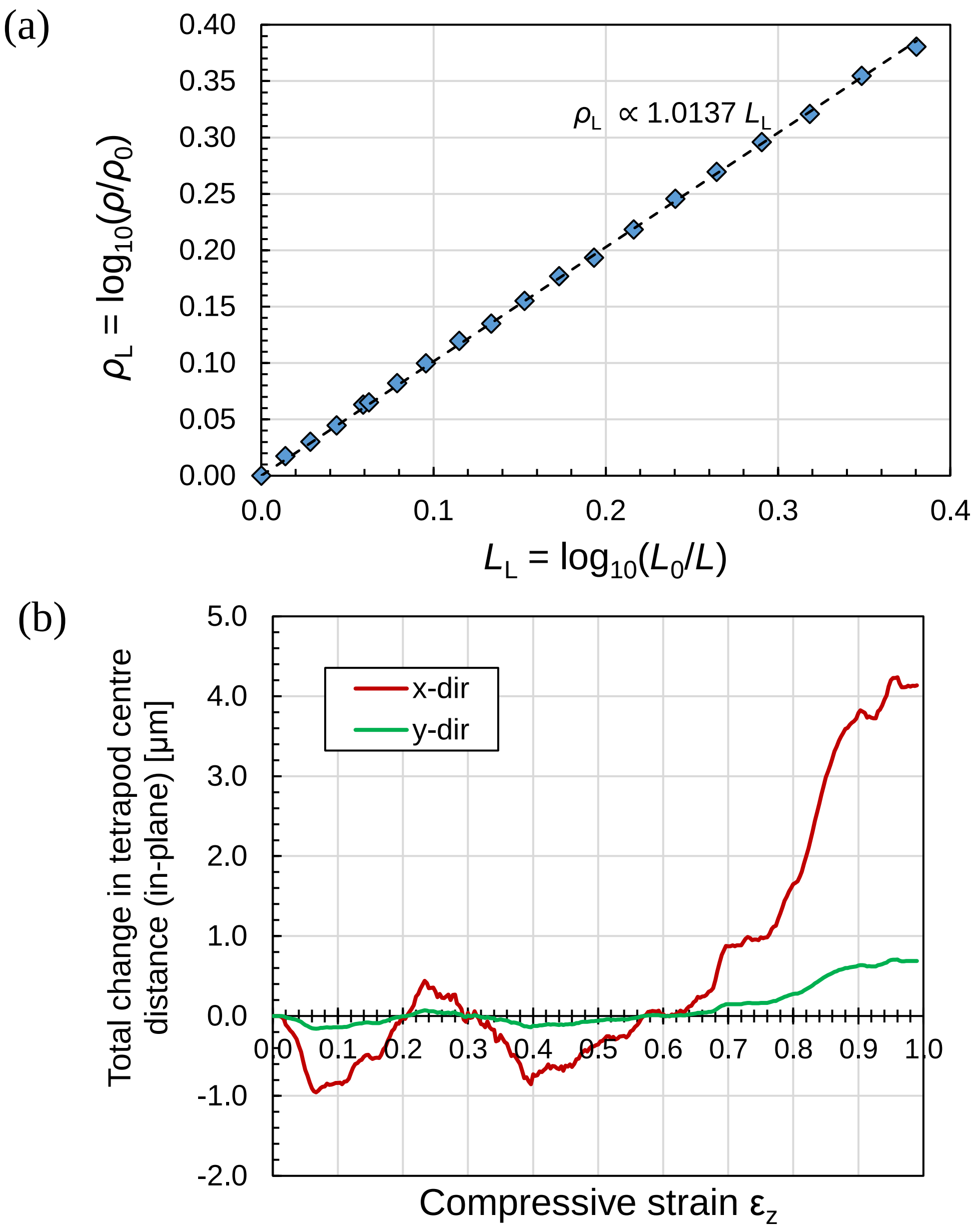

Supplementary Figure 24. Computation of the equivalent Poisson's ratio: a) Evolution of the tetrapod network density as a function of axial length from FEM simulations of monoaxial compression (cfr. with the theoretical prediction $\beta = 1\text{-}\upsilon$ in **Supplementary Table 2**, which provides $\nu = -0.014$). b) Total change in the tetrapod center distance, i.e. the sum of the changes in centre-to-centre distance of all tetrapods in the RVE (*x*- and *y*- directions, load applied in *z*-direction) as a function of the compressive strain. We consider 9 tetrapods having an initial minimum centre-to-centre distance ~ 20 µm (adjacent tetrapods) and a maximum distance ~ 44 µm (mutually farthest tetrapods). $\varepsilon_z = 1$ corresponds to a total vertical displacement of the load cell Δl ~ 44 µm. Results show a slightly negative Poisson's ratio (but not significantly different from zero) up to 60% compression, where the behavior is governed by the bending/buckling of arms.[41]

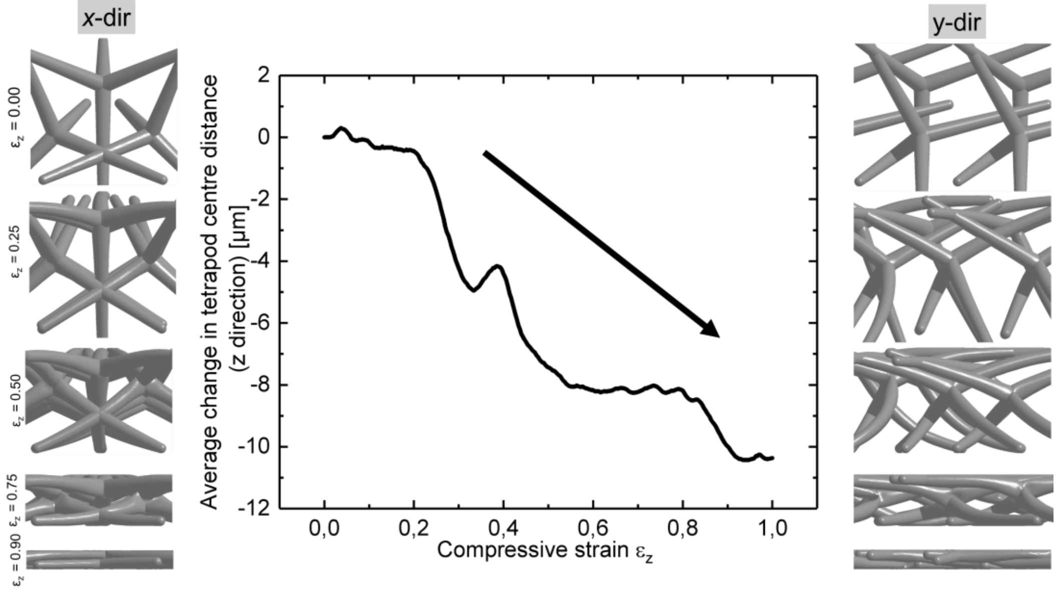

Supplementary Figure 25. Variation of tetrapod centre-to-centre distance by FEM simulations: The average tetrapod centre distance (between adjacent elements) in *z*-direction at the beginning is ~ 10 µm and reduces as the compressive strain increases. The pictures show snapshots of the simulated network (initial $\boldsymbol{\rho}_{\mathbf{Aero-BN}}$ ~ 0.376 mg cm^{-3}) at different compressive strains, giving a qualitative representation of the change in the distance between tetrapod centres and hollow tube arms.

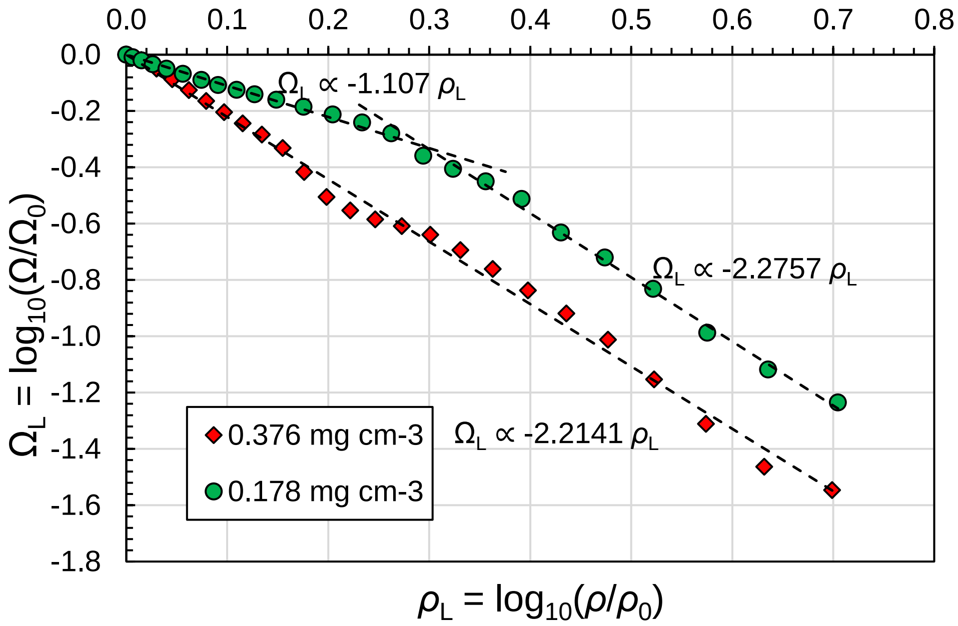

Supplementary Figure 26. Scaling of the projected porous area under monoaxial compression with the unit cell density for different initial Aero-BN network density from FEM simulations.

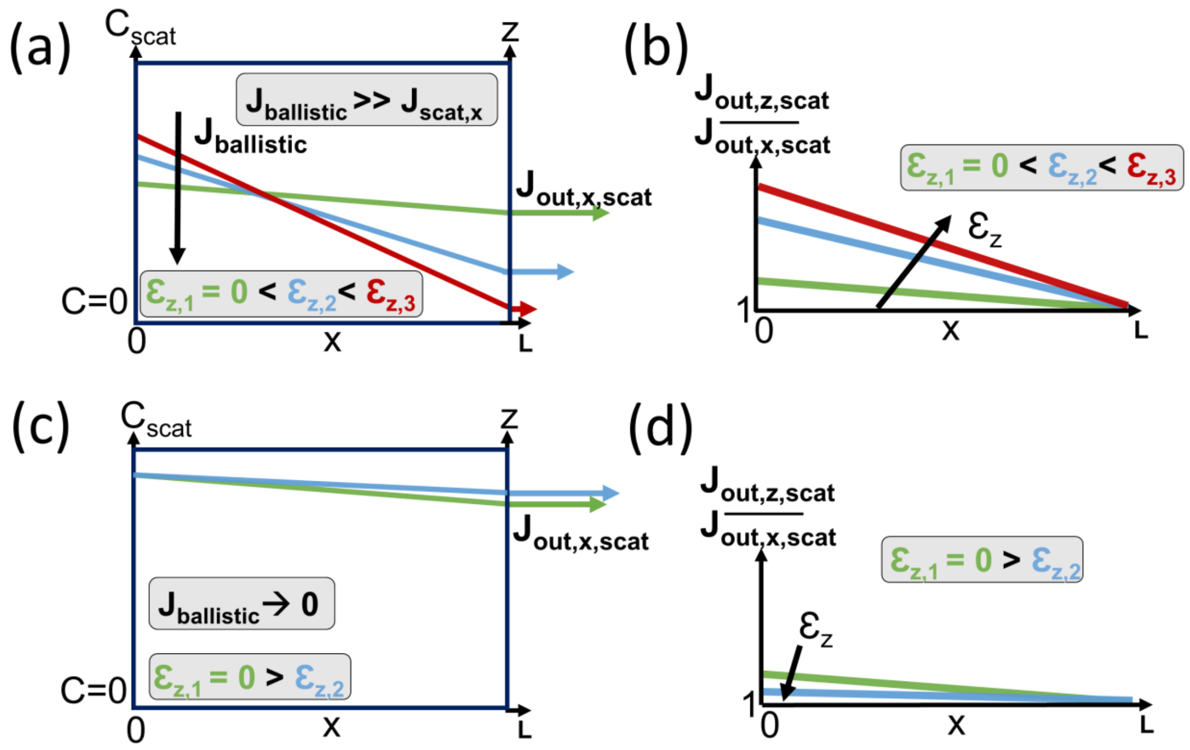

Supplementary Figure 27. Qualitative results of the proposed light transport model in Aero-BN: a,b) for low density Aero-BN samples ($\boldsymbol{\rho}_{\mathbf{Aero-BN}}$ < 0.57 mg cm^{-3}) as a function of compression and c,d) for high Aero-BN samples ($\boldsymbol{\rho}_{\mathbf{Aero-BN}}$ > 0.57 mg cm^{-3}) as a function of elongation.

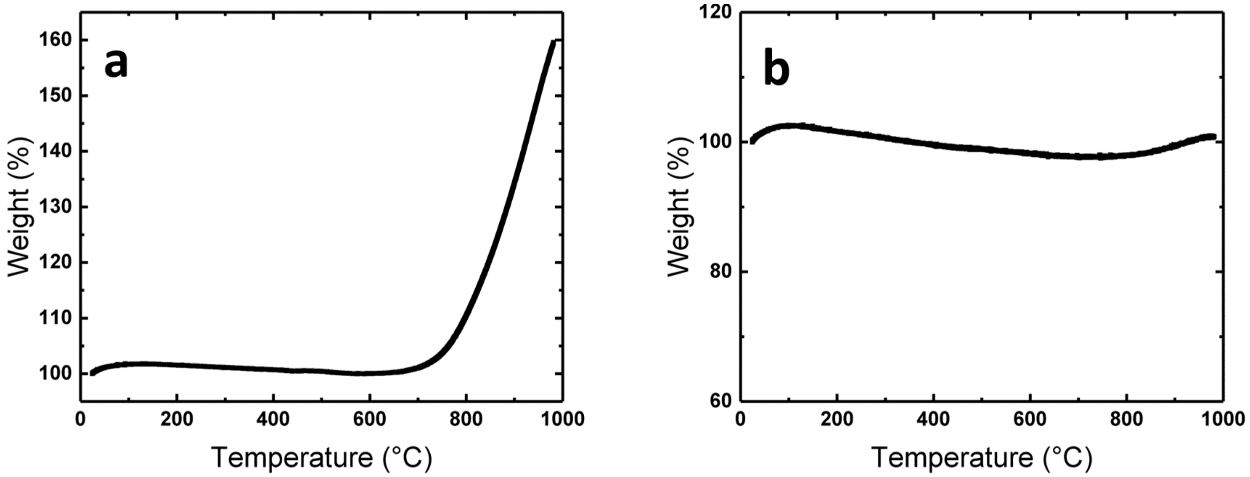

Supplementary Figure 28. TGA of Aero-BN under a) N_2 and O_2 (1:4) and b) pure N_2.

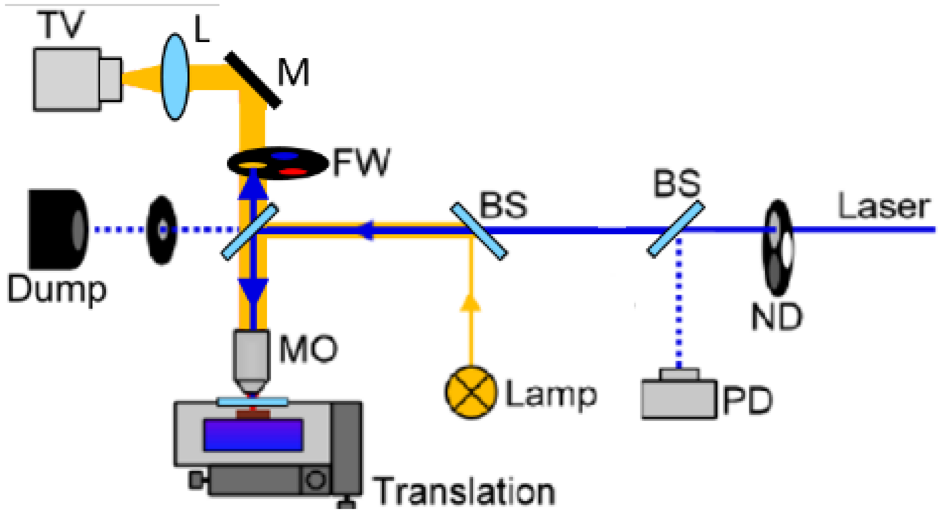

Supplementary Figure 29. Schematic setup for measurement of laser induced damage threshold: A 532 nm laser (100 Hz, ~ 7 ns pulse duration) is guided through an adjustable attenuator (ND), two beam splitters (BS), reflected by a third BS and focused on the sample by an objective (MO). The first split part of the laser light (dashed line) is directed onto a Si-diode power meter (PD) to measure the laser power during the measurements. The laser intensity on the sample is corrected afterwards by a calibration measurement (performed at the sample position). The white light (Lamp) for the microscopic imaging is coupled into the beam path by a BS and focused by MO. The backscattered white light is imaged using a video camera behind a mirror (M) and a focusing lens (L), while the laser is blocked by a filter wheel (FW).

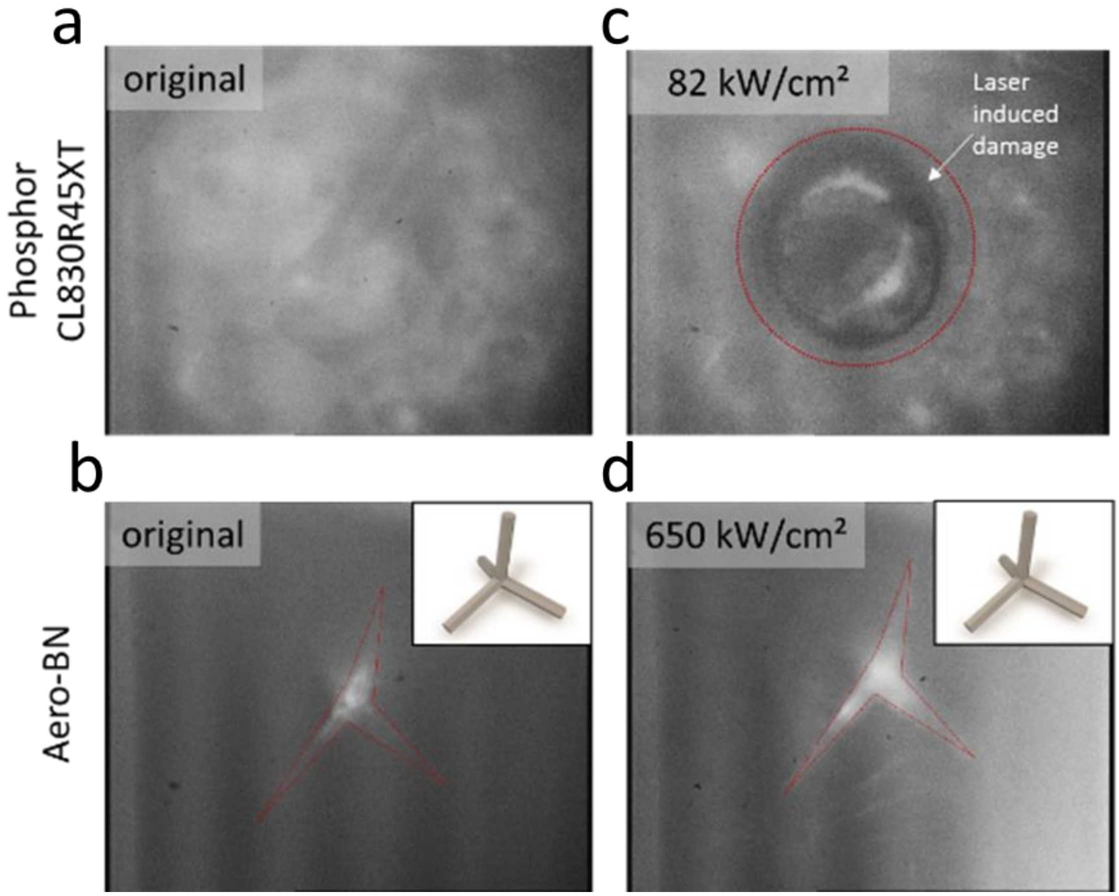

Supplementary Figure 30. Microscopy photographs of a commercial available remote phosphor and an Aero-BN network before (a,b) and after (c,d) 450 nm CW laser illumination, respectively. The laser is focused to a spot ~ 8.4 µm diameter using the setup shown in **Supplementary Figure 29** and the power is increased stepwise. At ~ 80 kW cm^{-2} the remote phosphor undergoes laser induced degradation (marked by the red circle), whereas the Aero-BN network structure remains intact even at high power densities (650 kW cm^{-2}). The red lines and the inset highlight the position of the illuminated Aero-BN microtubes.

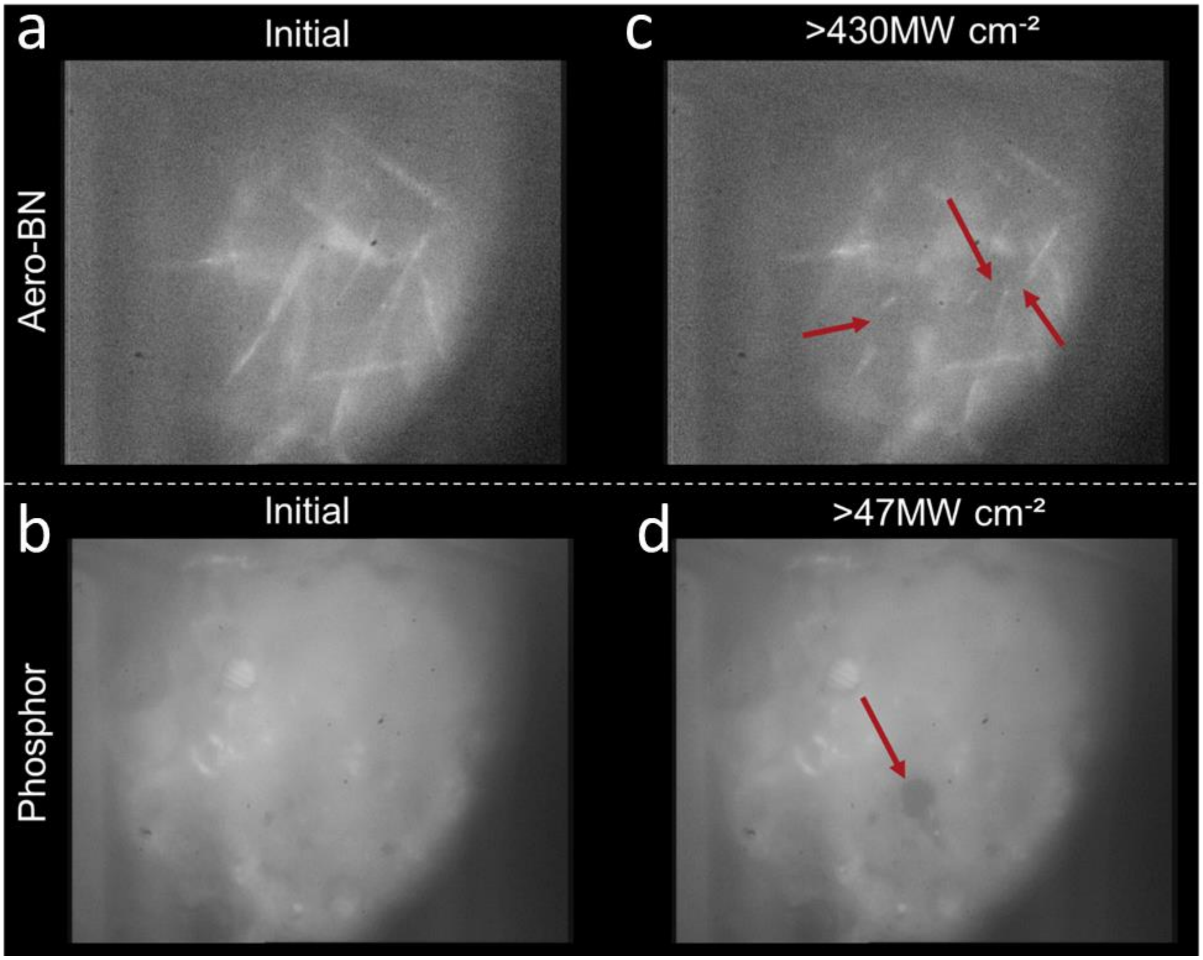

Supplementary Figure 31. Microscopy photographs of hollow Aero-BN microtube network and a phosphor (Intermatix CL830R45XT) before (a,b) and after (c,d) 355 nm illumination (100 Hz repetition rate, ~ 7 ns pulse duration). The laser spot is focused (spot diameter ~ 1 µm) and moved over the samples using the setup shown in **Supplementary Figure 29.** The power is increased stepwise. At elevated laser powers (> 430 MW cm^{-2}) the tubes are destroyed, whereas the phosphor undergoes destruction at ~ 47 MW cm^{-2}. The laser induced damage is indicated by the red arrows. The corresponding power is taken as the damage threshold.

Supplementary Note 7. Effect of temperature on speckle patterns

The low speckle contrasts (< 4 %) we obtain using the Aero-BN as an optical diffuser benefit from small (in the nm regime) thermally activated movements of the hollow microtubes. These movements can result in a time-varying speckle, similar to colloidal dispersions[46]. Since Aero-BN can withstand high temperatures (< 600 °C) without decomposition, we simulate the vibration of the microtube network as a function of temperature. The thin walls of the microtubes (< 25 nm thickness) can be modeled as beams. The tube, with average length r and moment of inertia $I = \frac{\pi}{8} d_{\mathrm{air}}^3 d_{\mathrm{wall}}$ as determined from our samples, is modeled with the Euler's beam formulation[47]. For an interconnected network the arms of different tetrapods are mutually entangled and they can be approximated by simply supported beams, at the central joint and at the connecting point with the other arms. According to this hypothesis on boundary conditions, the amplitude of the natural vibration of order n is given by[48]:

$$B_n^2 = \frac{4hfr^3}{n^4\pi^4(e^{hf/k_{\mathrm{b}}T} - 1)} \qquad \text{(S6)}$$

where h = 6.62607004·10^{-34} J·s is the Planck's constant, k_{b} = 1.3806485279·10^{-23} J K^{-1} is the Boltzmann's constant, T is the temperature at which the beam is subjected and $f = \omega_{\mathrm{n}}/2\pi$ is the n order natural frequency of the harmonic oscillator, depending on the static scheme[47]. Calculations are made assuming for the tapered tube an average diameter d_{air} = 4 µm corresponding to the value at r/2. In **Supplementary Table 4** the results for the first 5 modes of vibration at different T and their geometrical combinations are reported. The computed amplitude ranges from 13.4 nm at 300 K to 24.5 nm at 1000 K, which is the maximum T we consider, as at higher temperatures hBN will decompose, even in inert gas atmosphere. Even though the difference in amplitude between the two T is ~ 11.1 nm, this might have a strong effect on the speckle pattern, since these

movements are in the same order of magnitude as the Brownian movements in colloidal dispersions[46]. Thus, at temperatures above room temperature, the vibration of the microtubes will promote a decrease in speckle contrast reduction.

Supplementary Table 4. Results of modal analysis (amplitude) of tetrapod arms at different *T*.

T **[K]**	B_1 **[m]**	B_2 **[m]**	B_3 **[m]**	B_4 **[m]**	B_5 **[m]**	$B = \sqrt{\sum B_n^2}$ **[m]**
300	1.29E-11	3.23E-12	1.43E-12	8.06E-13	5.16E-13	**1.34E-11**
350	1.39E-11	3.48E-12	1.55E-12	8.71E-13	5.57E-13	**1.45E-11**
400	1.49E-11	3.72E-12	1.66E-12	9.31E-13	5.96E-13	**1.55E-11**
450	1.58E-11	3.95E-12	1.76E-12	9.88E-13	6.32E-13	**1.64E-11**
500	1.67E-11	4.16E-12	1.85E-12	1.04E-12	6.66E-13	**1.73E-11**
550	1.75E-11	4.37E-12	1.94E-12	1.09E-12	6.99E-13	**1.82E-11**
600	1.82E-11	4.56E-12	2.03E-12	1.14E-12	7.30E-13	**1.90E-11**
650	1.90E-11	4.75E-12	2.11E-12	1.19E-12	7.60E-13	**1.97E-11**
700	1.97E-11	4.93E-12	2.19E-12	1.23E-12	7.88E-13	**2.05E-11**
750	2.04E-11	5.10E-12	2.27E-12	1.28E-12	8.16E-13	**2.12E-11**
800	2.11E-11	5.27E-12	2.34E-12	1.32E-12	8.43E-13	**2.19E-11**
850	2.17E-11	5.43E-12	2.41E-12	1.36E-12	8.69E-13	**2.26E-11**
900	2.23E-11	5.59E-12	2.48E-12	1.40E-12	8.94E-13	**2.32E-11**
950	2.30E-11	5.74E-12	2.55E-12	1.44E-12	9.18E-13	**2.39E-11**
1000	2.36E-11	5.89E-12	2.62E-12	1.47E-12	9.42E-13	**2.45E-11**

Supplementary Note 8. Efficency considerations of Aero-BN lighting systems

Our approach of using a special designed hBN foam material as a diffuser for laser light indicates a viable alternative to the use of phosphorous materials by enabling a highly efficient (~ 98%) mixing of multi-colored laser light at very high powers (~ 430 MW cm^{-2}) and very low speckle (< 4%).

While in the case of phosphorous materials the overall efficiency is mainly determined by the efficiency of the used blue laser diode, as well as the conversion efficiency of the phosphorous material, the efficiency of our material is determined by the efficiency of the individual laser diodes used to produce blue, green and red light. Current state-of-the-art red laser diodes have shown efficiencies up to 40%[49,50], while efficiencies of ~20%[51] and ~40%[51,52] for green and blue respectively, were demonstrated. Nevertheless, continuous progress is being made with respect to the increase of laser diode efficiencies and several papers have indicated that the proposed LD efficiency (>50%) of laser diodes will in the near future approach that of LEDs, while providing a much higher current density. [4,52–54]

This increase in efficiency of blue lasers will also result in an increased efficiency of phosphorous materials. However, the phosphorous systems are strongly limited by their low irradiance levels (< 20 kW cm^{-2})[55], making them unsuitable for high brightness applications requiring light outputs of several MW cm^{-2}. Secondly, phosphorous materials are capable of providing only a certain color for illumination, while for the future lighting technology color tunability will be an important aspect, enabling new functionalities and designs in lighting.[53]

Therefore, our concept paves the way for a new generation of laser-based RGB light sources by introducing a diffuser with a negligible absorbtion and capable of reducing speckle contrast well-below the threshold of the human eye.

Supplementary References

1. Y. Kuratomi, K. Sekiya, H. Satoh, T. Tomiyama, T. Kawakami, B. Katagiri, Y. Suzuki & T. Uchida, *Speckle reduction mechanism in laser rear projection displays using a small moving diffuser*, *Journal of the Optical Society of America. A, Optics, image science, and vision* **27,** 1812–1817 (2010).

2. T. Alqurashi, P. Penchev, A. K. Yetisen, A. Sabouri, R. M. Ameen, S. Dimov & H. Butt, *Femtosecond laser directed fabrication of optical diffusers, RSC Adv* **7,** 18019–18023 (2017).

3. B. W. Lim & M. C. Suh, *Simple fabrication of a three-dimensional porous polymer film as a diffuser for organic light emitting diodes, Nanoscale* **6,** 14446–14452 (2014).

4. C. Basu, M. Meinhardt-Wollweber & B. Roth, *Lighting with laser diodes*, *Advanced Optical Technologies* **2** (2013).

5. H. Murata, K. Shibasaki, K. Yamamoto & Y. Okamura, *Speckle control using high-frequency signal superposition to semiconductor laser, OPT REV* **21,** 79–82 (2014).

6. T. Stangner, H. Zhang, T. Dahlberg, K. Wiklund & M. Andersson, *Step-by-step guide to reduce spatial coherence of laser light using a rotating ground glass diffuser*, *Appl. Opt.* **56,** 5427 (2017).

7. W. Thomas & C. Middlebrook, *Non-moving Hadamard matrix diffusers for speckle reduction in laser pico-projectors*, *Journal of modern optics* **61,** S74-S80 (2014).

8. W. Lei, V. N. Mochalin, D. Liu, S. Qin, Y. Gogotsi & Y. Chen, *Boron nitride colloidal solutions, ultralight aerogels and freestanding membranes through one-step exfoliation and functionalization*, *Nature communications* **6,** 8849 (2015).

9. M. Loeblein, S. H. Tsang, M. Pawlik, E. J. R. Phua, H. Yong, X. W. Zhang, C. L. Gan & E. H. T. Teo, *High-Density 3D-Boron Nitride and 3D-Graphene for High-Performance Nano-Thermal Interface Material, ACS nano* **11,** 2033–2044 (2017).

10. P. S. Owuor, O.-K. Park, C. F. Woellner, A. S. Jalilov, S. Susarla, J. Joyner, S. Ozden, L. Duy, R. Villegas Salvatierra, R. Vajtai, J. M. Tour, J. Lou, D. S. Galvão, C. S. Tiwary & P. M. Ajayan, *Lightweight Hexagonal Boron Nitride Foam for CO2 Absorption, ACS nano* (2017).

11. Y. Song, B. Li, S. Yang, G. Ding, C. Zhang & X. Xie, *Ultralight boron nitride aerogels via template-assisted chemical vapor deposition, Scientific reports* **5,** 10337 (2015).

12. Y. Xue, P. Dai, M. Zhou, X. Wang, A. Pakdel, C. Zhang, Q. Weng, T. Takei, X. Fu, Z. I. Popov, P. B. Sorokin, C. Tang, K. Shimamura, Y. Bando & D. Golberg, *Multifunctional Superelastic Foam-Like Boron Nitride Nanotubular Cellular-Network Architectures, ACS nano* **11,** 558–568 (2017).

13. J. Yin, X. Li, J. Zhou & W. Guo, *Ultralight three-dimensional boron nitride foam with ultralow permittivity and superelasticity, Nano letters* **13,** 3232–3236 (2013).

14. A. W. Searcy & C. E. Myers, *The Heat of Sublimation of Boron and the Gaseous Species of the Boron–Boric Oxide System, J. Phys. Chem.* **61,** 957–960 (1957).

15. D. E. Harrison & F. A. Hummel, *Phase Equilibria and Fluorescence in the System Zinc Oxide-Boric Oxide, J. Electrochem. Soc.* **103,** 491 (1956).

16. P. W. Atkins, J. de Paula & M. Bär. *Physikalische Chemie.* 5th ed. (Wiley-VCH Verl., Weinheim, 2013).

17. P. M. Schaber, J. Colson, S. Higgins, D. Thielen, B. Anspach & J. Brauer, *Thermal decomposition (pyrolysis) of urea in an open reaction vessel, Thermochimica Acta* **424,** 131–142 (2004).

18. M. Mesrine, N. Grandjean & J. Massies, *Efficiency of NH3 as nitrogen source for GaN molecular beam epitaxy, Appl. Phys. Lett.* **72,** 350–352 (1998).

19. M. Mecklenburg, A. Schuchardt, Y. K. Mishra, S. Kaps, R. Adelung, A. Lotnyk, L. Kienle & K. Schulte, *Aerographite: ultra lightweight, flexible nanowall, carbon microtube material with outstanding mechanical performance, Advanced materials* **24,** 3486–3490 (2012).

20. J. Marx, D. Smazna, R. Adelung & B. Fiedler, *Growth Model of a 3d Carbon Structure (Aerographite) in a Replica-Chemical Vapor Deposition Process and Its Properties, World Academy of Science, Engineering and Technology, International Journal of Materials and Metallurgical Engineering* **5** (2018).

21. Y. K. Mishra & R. Adelung, *ZnO tetrapod materials for functional applications, Materials Today* **21,** 631–651 (2017).

22. Z. Chen, W. Ren, L. Gao, B. Liu, S. Pei & H.-M. Cheng, *Three-dimensional flexible and conductive interconnected graphene networks grown by chemical vapour deposition*, *Nature materials* **10,** 424–428 (2011).

23. C. Souche, B. Jouffrey, G. Hug & M. Nelhiebel, *Orientation Sensitive EELS-analysis of Boron Nitride Nanometric Hollow Spheres*, *Micron* **29,** 419–424 (1998).

24. T. Hemraj-Benny, S. Banerjee, S. Sambasivan, M. Balasubramanian, D. A. Fischer, G. Eres, A. A. Puretzky, D. B. Geohegan, D. H. Lowndes, W. Han, J. A. Misewich & S. S. Wong, *Near-edge X-ray absorption fine structure spectroscopy as a tool for investigating nanomaterials*, *Small (Weinheim an der Bergstrasse, Germany)* **2,** 26–35 (2006).

25. J. Moscovici, G. Loupias, P. H. Parent & G. Tourillon, *Polarization-dependent boron and nitrogen K nexafs of hexagonal BN*, *Journal of Physics and Chemistry of Solids* **57,** 1159–1161 (1996).

26. H. C. Choi, S. Y. Bae, W. S. Jang, J. Park, H. J. Song & H.-J. Shin, *X-ray absorption near edge structure study of BN nanotubes and nanothorns*, *The journal of physical chemistry. B* **109,** 7007–7011 (2005).

27. D. Usachov, V. K. Adamchuk, D. Haberer, A. Grüneis, H. Sachdev, A. B. Preobrajenski, C. Laubschat & D. V. Vyalikh, *Quasifreestanding single-layer hexagonal boron nitride as a substrate for graphene synthesis*, *Phys. Rev. B* **82** (2010).

28. Y. Wang & Y. Ding, *Tunable magnetic and electronic properties of BN nanosheets with triangular defects: a first-principles study*, *Journal of physics. Condensed matter : an Institute of Physics journal* **26,** 435302 (2014).

29. R. Kilaas, *Optimal and near-optimal filters in high-resolution electron microscopy*, *J Microsc* **190,** 45–51 (1998).

30. T. Malis, S. C. Cheng & R. F. Egerton, *EELS log-ratio technique for specimen-thickness measurement in the TEM*, *Journal of electron microscopy technique* **8,** 193–200 (1988).

31. G. Karlsson, *Thickness measurements of lacey carbon films*, *J Microsc* **203,** 326–328 (2001).

32. P. Wang, W. Körner, A. Emmerling, A. Beck, J. Kuhn & J. Fricke, *Optical investigations of silica aerogels*, *Journal of Non-Crystalline Solids* **145,** 141–145 (1992).

33. L. Zhao, S. Yang, B. Bhatia, E. Strobach & E. N. Wang, *Modeling silica aerogel optical performance by determining its radiative properties*, *AIP Advances* **6,** 25123 (2016).

34. N. Hüsing & U. Schubert, *Aerogels—Airy Materials:* Chemistry, Structure, and Properties, *Angewandte Chemie International Edition* **37,** 22–45 (1998).

35. A. Emmerling, R. Petricevic, A. Beck, P. Wang, H. Scheller & J. Fricke, *Relationship between optical transparency and nanostructural features of silica aerogels*, *Journal of Non-Crystalline Solids* **185,** 240–248 (1995).

36. J. L. Gurav, I.-K. Jung, H.-H. Park, E. S. Kang & D. Y. Nadargi, *Silica Aerogel:* Synthesis and Applications, *Journal of Nanomaterials* **2010,** 1–11 (2010).

37. *Disordered times*, *Nature Photon* **7,** 161 (2013).

38. K. J. Pascoe. *Reflectivity and transmissivity through layered, lossy media. A user-friendly approach* (Biblioscholar, 2012).

39. H. C. van de Hulst. *Light Scattering by Small Particles* (Dover Publications, Newburyport, 2012).

40. K. Shehzad, Y. Xu, C. Gao & X. Duan, *Three-dimensional macro-structures of two-dimensional nanomaterials*, *Chemical Society reviews* **45,** 5541–5588 (2016).

41. R. Meija, S. Signetti, A. Schuchardt, K. Meurisch, D. Smazna, M. Mecklenburg, K. Schulte, D. Erts, O. Lupan, B. Fiedler, Y. K. Mishra, R. Adelung & N. M. Pugno, *Nanomechanics of individual aerographite tetrapods*, *Nature communications* **8,** 14982 (2017).

42. D. S. Wiersma, P. Bartolini, A. Lagendijk & R. Righini, *Localization of light in a disordered medium*, *Nature* **390,** 671–673 (1997).

43. S. Mohan, H. Ramachandran & N. Kumar, *Localization of light by magnetically tuned correlated disorder:* Trapping of light in ferrofluids (2009).

44. R. Schittny, A. Niemeyer, F. Mayer, A. Naber, M. Kadic & M. Wegener, *Invisibility cloaking in light-scattering media*, *Laser & Photonics Reviews* **10,** 382–408 (2016).

45. M. F. Beatty & D. O. Stalnaker, *The Poisson Function of Finite Elasticity*, *J. Appl. Mech.* **53,** 807 (1986).

46. F. Riechert, G. Bastian & U. Lemmer, *Laser speckle reduction via colloidal-dispersion-filled projection screens*, *Appl. Opt.* **48,** 3742 (2009).

47. S. P. Timošenko & J. N. Goodier. *Theory of elasticity.* 3rd ed. (McGraw-Hill, New York, NY, 1970).

48. L. Wang & H. Hu, *Thermal vibration of single-walled carbon nanotubes with quantum effects*, *Proceedings. Mathematical, physical, and engineering sciences* **470,** 20140087 (2014).

49. B. Sumpf, M. Zorn, M. Maiwald, R. Staske, J. Fricke, P. Ressel, G. Erbert, M. Weyers & G. Trankle, *5.6-W Broad-Area Lasers With a Vertical Far-Field Angle of 31$^{\\circ}$ Emitting at 670 nm*, *IEEE Photon. Technol. Lett.* **20,** 575–577 (2008).

50. B. Sumpf, M. Zorn, R. Staske, J. Fricke, P. Ressel, A. Ginolas, K. Paschke, G. Erbert, M. Weyers & G. Trankle, *3-W Broad Area Lasers and 12-W Bars With Conversion Efficiencies up to 40% at 650 nm*, *IEEE J. Select. Topics Quantum Electron.* **13,** 1188–1193 (2007).

51. M. Murayama, Y. Nakayama, K. Yamazaki, Y. Hoshina, H. Watanabe, N. Fuutagawa, H. Kawanishi, T. Uemura & H. Narui, *Watt-Class Green (530 nm) and Blue (465 nm) Laser Diodes*, *Phys. Status Solidi A* **215,** 1700513 (2018).

52. J. J. Wierer, J. Y. Tsao & D. S. Sizov, *Comparison between blue lasers and light-emitting diodes for future solid-state lighting*, *Laser & Photonics Reviews* **7,** 963–993 (2013).

53. J. M. Phillips, M. E. Coltrin, M. H. Crawford, A. J. Fischer, M. R. Krames, R. Mueller-Mach, G. O. Mueller, Y. Ohno, L.E.S. Rohwer, J. A. Simmons & J. Y. Tsao, *Research challenges to ultra-efficient inorganic solid-state lighting*, *Laser & Photonics Reviews* **1,** 307–333 (2007).

54. A. Neumann, J. J. Wierer, W. Davis, Y. Ohno, S. R. J. Brueck & J. Y. Tsao, *Four-color laser white illuminant demonstrating high color-rendering quality*, *Optics express* **19,** A982-90 (2011).

55. N. Trivellin, M. Yushchenko, M. Buffolo, C. de Santi, M. Meneghini, G. Meneghesso & E. Zanoni, *Laser-Based Lighting:* Experimental Analysis and Perspectives, *Materials (Basel, Switzerland)* **10** (2017).
