# Supplementary material for: Conversionless efficient and broadband laser light diffusers for high brightness illumination applications
Source: Nat Commun. 2020 Mar 18;11:1437. doi: 10.1038/s41467-020-14875-z (PMC7080714; doi:10.1038/s41467-020-14875-z)
Supplement: Supplementary file 2 — Description of Additional Supplementary Files [file 41467_2020_14875_MOESM2_ESM.pdf]

Description of Additional Supplementary Files

File name: Supplementary Movie 1

Description: Tunable RGB laser-light room illumination using a macroscopic network of interconnected hollow hBN microtubes.
